# Photocrosslinkable natural polymers in tissue engineering

**DOI:** 10.3389/fbioe.2023.1127757

**Published:** 2023-03-02

**Authors:** Seo Hyung Moon, Hye Jin Hwang, Hye Ryeong Jeon, Sol Ji Park, In Sun Bae, Yun Jung Yang

**Affiliations:** ^1^ Department of Biological Sciences and Bioengineering, Inha University, Incheon, Republic of Korea; ^2^ Department of Biological Engineering, Inha University, Incheon, Republic of Korea

**Keywords:** photo-reactive moiety, photo-crosslinking, photoinitiator, catalyst, polymerization

## Abstract

Natural polymers have been widely used in scaffolds for tissue engineering due to their superior biocompatibility, biodegradability, and low cytotoxicity compared to synthetic polymers. Despite these advantages, there remain drawbacks such as unsatisfying mechanical properties or low processability, which hinder natural tissue substitution. Several non-covalent or covalent crosslinking methods induced by chemicals, temperatures, pH, or light sources have been suggested to overcome these limitations. Among them, light-assisted crosslinking has been considered as a promising strategy for fabricating microstructures of scaffolds. This is due to the merits of non-invasiveness, relatively high crosslinking efficiency *via* light penetration, and easily controllable parameters, including light intensity or exposure time. This review focuses on photo-reactive moieties and their reaction mechanisms, which are widely exploited along with natural polymer and its tissue engineering applications.

## 1 Introduction

Artificial scaffold has been engineered to mimic the spatial dimension of the extracellular matrix (ECM). This is because the 3D network affects the diffusion and release kinetics of biomolecules as well as the mechanical modulus and rheological properties of the whole scaffold (Ko et al., 2010; Pei et al., 2019). The macro/microstructure of the scaffold can be tailored by chemical or physical crosslinking with a suitable choice of reactive moieties (Mihaila et al., 2012; Marizza et al., 2016).

While the physical intramolecular interactions involve weak non-covalent interactions, the chemical crosslinking is not transient due to covalent interactions (Marizza et al., 2016; Samani et al., 2020). In most cases, the stimulus of chemical crosslinking is a chemical agent or light source. Remarkably, light-initiated photocrosslinking has attracted attention due to its easily controllable parameters such as light intensity, exposure time, or irradiation distance (Chandrasekharan et al., 2019; Choi and Cha, 2019; Yano et al., 2020). Most of all, the non-invasiveness and capability of *in situ* photopolymerizations represent the unique and powerful merits of light-initiated photocrosslinking.

The polymerization between the functional groups of polymer chains activated by light exposure with or without photoinitiators is called photocrosslinking (Smeds et al., 2001). During this process, highly reactive free radicals are generated by photocleavage. The photoinitiators absorb light photons and convert light energy into chemical energy (Maiz-Fernández et al., 2022). The free radicals are covalently crosslinked with intra- or extra-molecular groups. Tyrosine/tyramine and methacryloyl are the most widely used photo-responsive moieties of biomaterials, followed by cinnamoyl, benzophenone, norbornene, aryl azide, and diazirine.

Photo-reactions triggered by functional groups activation facilitate bulk polymer crosslinking, photo-immobilization, surface modification, molecular labeling, or particle fabrication (Fernández and Orozco, 2021). These are applicable to both two-dimensional and three-dimensional structures (spatially addressable effects) with high selectivity and efficiency without producing toxic or reactive side products (Kim et al., 2017). It is important to select the appropriate photo-reactive moiety of natural polymer derivatives which meet the characteristics of the purposed applications in tissue engineering.

As well known, numerous reviews have covered the crosslinked natural polymers utilized in biomedical applications. In this review, photo-reactive or photo-responsive moieties and their reaction mechanisms were covered, which are widely exploited along with natural polymer and its tissue engineering applications. The characteristics of reactions, the chemical substitution of functional groups on target biopolymers, and types of photoinitiators depending on the light source for each moiety are discussed. Additionally, recent studies on each photopolymerization technique are introduced to enhance readers’ understanding.

### 1.1 Tyrosine and tyramine

Tyrosine is a reactive amino acid with a phenolic ring that can easily be transformed into hydrogels with or without chemical reagents *via* phenolic oxidation (Sogawa et al., 2020; Liu et al., 2021). The phenolic functional group of tyrosine contributes to the π-π interaction in terms of structural stability and the proton-coupled electron transfer reactions in energy transportation. The phenolic side chain allows crucial biosynthesis in nature systems by forming dityrosine crosslinks *via* electron transfer (Raven et al., 1971; Partlow et al., 2016; Lee et al., 2019). The tyrosine-tyrosine chemical bonds provide elasticity to biomaterials, as seen in diverse organisms. Examples of elastic biomaterials are the wing tendon of dragonflies (the protein named resilin), the cuticles of locus, and the silk fibroin of silkworms (Partlow et al., 2016). The covalent bonds within dityrosine can be emulated *in vitro via* enzymes or photo-based radical reactions to attain the physical and functional properties of polymeric biomaterials (Partlow et al., 2016).

These phenolic crosslinking systems are not only possible in tyrosine rich-natural proteins or peptides, but also in tyramine-modified materials including, polysaccharides (alginate, hyaluronic acid, dextran, *etc.*) or synthetic polymers (poly (ethylene glycol), poly (vinyl alcohol), *etc.*) (Roberts et al., 2016). As mentioned previously, the *in vitro* dityrosine crosslink has been employed to increase the mechanical properties of biomaterials containing silk fibroin (5.3% tyrosine) (Gong et al., 2020; Mu et al., 2020; Huang Y. et al., 2022), keratin (22% tyrosine) (Gillespie, 1972; Sando et al., 2010; Navarro et al., 2018), fibrinogen (5.6% tyrosine in γ-chain; 4.9% tyrosine in β-chain; .65% tyrosine in α-chain) (Elvin et al., 2009), marine-derived silk (aneroin, 5% tyrosine) (Park et al., 2019), and recombinant resilin (*rec1*-resilin, 6% tyrosine) (Truong et al., 2011). In order to increase the mechanical durability of polysaccharides (alginate, hyaluronic acid, *etc.*), the phenolic groups have been introduced through tyramine conjugation, synthesized by the EDC/NHS [N-(3-dimethylaminopropyl)-N′-ethylcarbodiimide hydrochloride/N-hydroxysuccinimide] chemistry (Schulz et al., 2019; Wang et al., 2020; Kim E. et al., 2021). The crosslinking density is also adjustable with diverse photoinitiator concentrations and photo-irradiation intensities (distance- and time-dependent).

**FIGURE 1:**
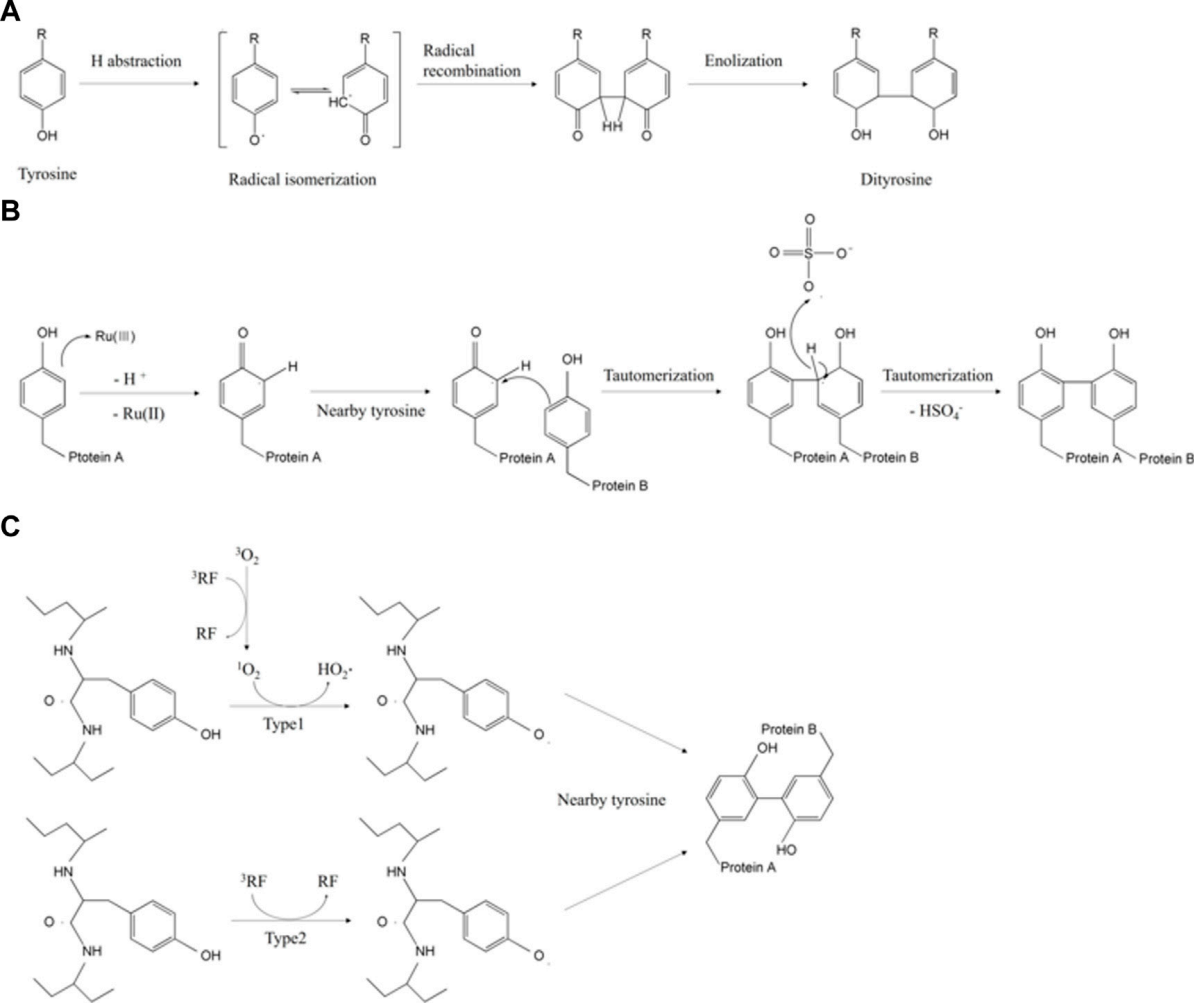
Overview of tyrosyl residue crosslinking mechanisms initiated by **(A)** UV light (Correia et al., 2012), **(B)** Ru (Ⅱ) polypyridine (Fancy and Kodadek, 1999), and **(C)** riboflavin (Liu et al., 2021).

Among the diverse dityrosine crosslinking triggers, photo-based crosslinking has significant advantages in terms of non-invasiveness, fast reaction rate, fine tunability, and crosslinking efficiency (Choi and Cha, 2019; Mu et al., 2020; Perin et al., 2022). The light source can be ultraviolet (UV) or visible light. UV irradiation generates radical cations (Tyr-OH·^+^) and solvated electrons to form tyrosyl radicals. These radicals induce continuous polymerization between tyrosine-tyrosine residues *via* deprotonation, radical isomerization, diradical production, and enolization (Figure 1A) (Houée-Lévin et al., 2015; Min et al., 2016). This one-step UV-mediated dityrosine crosslink is a covalent self-assembly system within short peptides and does not require additives (Min et al., 2016). Dityrosine units in tyrosine-rich peptides or other biomaterials function as not only stable assembly motifs but also multifunctional templates which allows self-assembled structure of organic/inorganic hybrid biomaterials to feature its chemical, electrochemical and structural properties triggered by the proton-coupled electron-transfer reactions (Lee et al., 2019). However, UV irradiation also regulates the antibody binding capability of peptide hormones, and *in vitro* hormonal function which could be exploited in pharmaceutical industry to estimate hormone’s structure and bioactivity (Correia et al., 2012). For example, the continuous UV excitation induced structural changes by forming tyrosine photo-product dityrosine, leading to covalent insulin dimerization and decreased antibody binding affinity up to 62.1% when irradiated 276 nm for 3.5 h (Correia et al., 2012).

As for the visible light system, ruthenium (Ⅱ) polypyridine [Ru (Ⅱ) polypyridine] and persulfate are essentially needed. Ru (Ⅱ) polypyridine generates electrons under visible light. The generated electrons are transferred to the persulfate acceptor. At the same time, tyrosine radicals are oxidated and crosslinked into dityrosine (Figure 1B) (Fancy and Kodadek, 1999). Here, the ruthenium complexes have higher excitation selectivity, a relatively longer excited state period, and higher chemical stability than other photoinitiators (Kim H. et al., 2021). Thus, many previous works of literature on silk fibroin, collagen, gelatin, and recombinant resilin have exploited ruthenium-mediated photo-crosslinking (Liu et al., 2021). In one study, the tyramine-modified gelatin methacrylate (GelMA-Tyr) hydrogels were photo-irradiated by visible light with Ru/SPS initiators for cartilage-binding glue (Lim et al., 2020a). Its adhesion strength (13.25 kPa) was 15 times higher than that of UV(365 nm)-crosslinked GelMA/Irgacure 2959 hydrogels because of the number of residues involved in photo-crosslinks (Lim et al., 2020a).

Under UV and visible light, riboflavin (vitamin B2) and its derivatives activate photo-catalysis by changing its state into single- or triplet-excited riboflavin (Huang et al., 2004). The single riboflavin generates oxygen intermediates and oxidizes tyrosine (Daood et al., 2013). Equivalently, the triplet riboflavin produces tyrosyl radicals, which form dityrosine linkages (Figure 1C) (Liu et al., 2021). In the presence of riboflavin, the silk fibroin gel produced by the photolithography can achieve ∼50 μm resolution due to fast photo-reactivity. The sophisticated fabrication with non-toxic photoinitiators and its own transparency has allowed elastic fibroin gel to be applied to ocular prostheses (Applegate et al., 2016).

For synergetic enhancement in mechanical properties and complexity of scaffolds, dual crosslinking is another strategy. In one research, the photo-crosslinked alginate-tyramine microfibers were further crosslinked ionically (Lim et al., 2021). The photo-crosslinking resulted in fast gelation within a few seconds, allowing the polysaccharide inks to be stacked layer by layer. The following ionic crosslinking was also beneficial to structural integrity (Park et al., 2019). Thermal and light-induced crosslinking method was used to fabricate 3D cell-laden scaffolds with decellularized extracellular matrix (dECM)-based bioinks at the centimeter scale with high printability where the minimal strand width was 100 μm (Kim H. et al., 2021). This dual-crosslinked product not only achieved 3.79 and 20.04-folds increases in elastic and resilient modulus respectively compared to of which the only thermo-physically crosslinked dECM products, but also have the ability of working as a functional tissue (Kim H. et al., 2021).

Overall, the dityrosine photo-crosslinking allows fabricating tissue mimicking polymeric materials due to its rapid gelation with improved homogeneity of network and superior mechanical integrity which meets the sufficient range of targeted elastic moduli (Camp et al., 2020; Liu et al., 2021). Besides, distinctive properties of UV-excited dityrosine crosslinking facilitate to regulate of the peptide hormones’ bioactivity and act as a novel bioreactor or probe for fluorescence detection of functional nanomaterials by UV-excited inherent blue fluorescence which is exploited in medical and pharmaceutical engineering (Correia et al., 2012; Min et al., 2018; Mukherjee et al., 2019).

### 1.2 Methacryloyl

Methacrylates and their derivatives have been extensively manipulated for light-activated crosslinking (Reis et al., 2009; Mu et al., 2020). Under UV conditions, the methacryloyl side chain generates free radicals, which rapidly crosslink each other. Methacrylate networks are not sensitive to the surrounding environmental conditions such as pH and temperature allowing good stability (Liu et al., 2022b). The degree of crosslinking is dependent on the number of methacryloyl substitutions (Kim S. H. et al., 2021).

Unfortunately, methacryloyl is not an intrinsic moiety of natural polymers, however, diverse methacrylating agents allow modify the chemical structures of the wide range of biomolecules and biopolymers including proteins, polysaccharides, synthetic polymers, as well as nanoparticles with high substitution yields depend on their functional groups (Samani et al., 2020). Methacrylates substitute the specific reactive residues of amine (-NH_2_), carboxyl (-COOH), and hydroxyl (-OH) groups, which are adjusted based on the reaction temperature, pH, and agitation speed (Chou and Nicoll, 2009; Yue et al., 2017). Therefore, the final product methacryloyl-modified biomaterials should be characterized by production conditions to confirm reproducibility regarding purity, methacrylation degree, and physiochemical properties as commercial utilization (Hasany et al., 2021).

**FIGURE 2:**
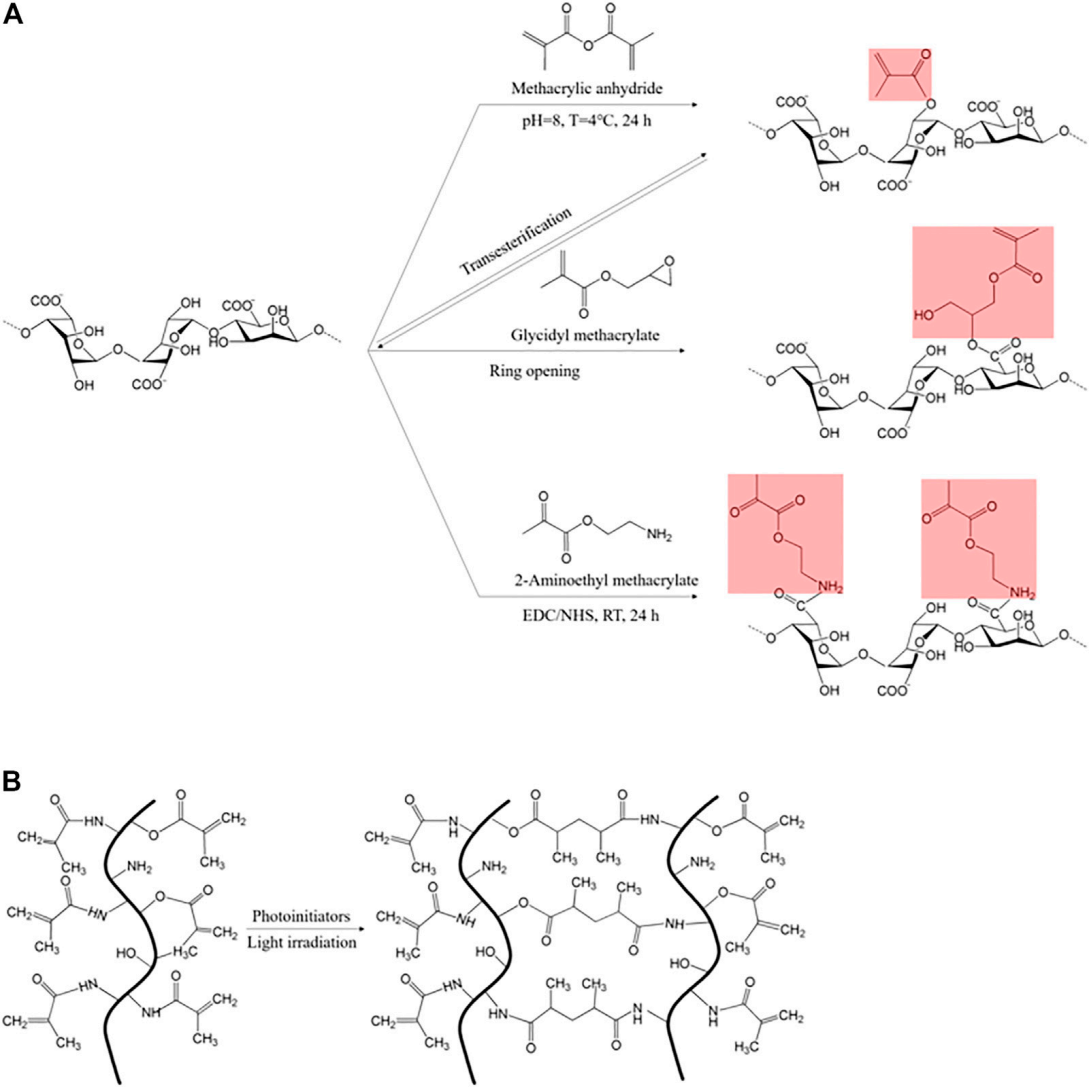
**(A)** Diverse methacryloyl substitution in methacrylic alginate induced by methacrylic anhydride, glycidyl methacrylate, and 2-aminoethyl methacrylate (Hasany et al., 2021), and **(B)** light-activated chain polymerization that occurred between two methacryloyl moieties (red: photo-reactive residues) (Bupphathong et al., 2022).

Methacrylic anhydride (MA) and glycidyl methacrylate (GMA) are commonly used reagents to introduce methacryloyl groups. Modification with MA is the most cost-effective and straightforward method for methacryloyl incorporation (Hasany et al., 2021). MA has a carbon-carbon double bond which readily undergoes free-radical polymerization and esterification with all three functional groups (-NH_2_, -COOH, and–OH) (Figure 2A). Therefore, it is widely utilized in various biopolymers, including silk, gelatin, hyaluronic acid, chitosan, alginate, starch, and pectin (Messager et al., 2013; Pereira et al., 2018b; Han et al., 2020; Noè et al., 2020; He et al., 2021). In some cases, GMA utilization is more plausible due to the problematic assessability of MA in the target material, such as silk, which has high crystallinity in acidic conditions (Mu et al., 2020). GMA is capable of epoxide ring-opening that facilitates methacrylate modification, even in acidic conditions. Additionally, the epoxide ring-opening reaction is processed predominantly over transesterification in acidic conditions (Li et al., 2003, 2017; Hasany et al., 2021). In addition to MA and GMA, AEMA (2-aminoethyl methacrylate) activates the carboxyl groups (-COOH) in hyaluronic acid or alginate under EDC/HNS reaction (Jeon et al., 2009).

**FIGURE 3:**
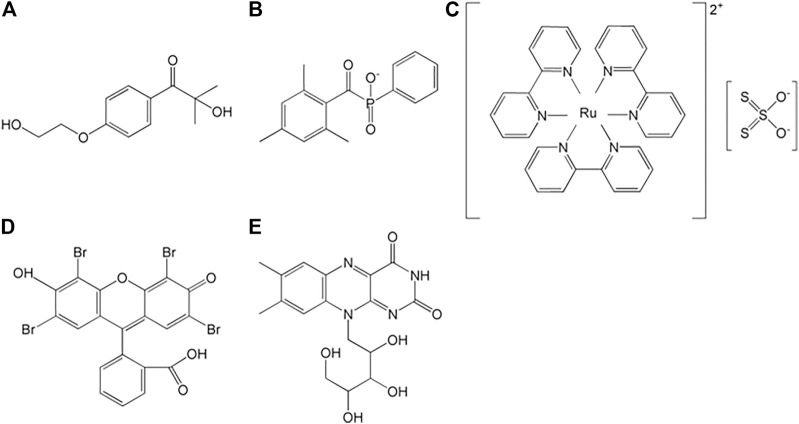
Common photoinitiators: **(A)** Irgacure 2959, **(B)** lithium phenyl-2,4,6-trimethylbenzoylphosphinate, **(C)** ruthenium (Ⅱ)/persulfate, **(D)** eosin Y, and **(E)** riboflavin (Mu et al., 2020).

The methacryloyl-initiated photocrosslinking is activated by diverse photoinitiators (Figure 3). Irgacure 2959 (2-hydroxy-1-[4-(2-hydroxyethoxy) phenyl]-2-methyl-1-propanone) and LAP (lithium acylphosphinate salt) generate free radicals under UV (365 and 405 nm, respectively) light, and simultaneously participate in chain polymerization, which occurs between two methacryloyl moieties (Figure 2B **)** (Basara et al., 2019). Tris (2,20-bipyridyl) dichlororuthenium (II) [Ru(II)] and eosin Y also induce photo-activated crosslinking under visible light, where their ranges are 420–450 and 450–550 nm, respectively (Noshadi et al., 2017; Lim et al., 2020a).

Gelatin-methacrylate (GelMA) is the first methacryloyl-modified form developed using GMA in the 1990s; since then, there have been many different methacryloyl-modified biomaterials, such as silk fibroin-methacrylate (SilMA), methacrylate hyaluronic acid (HAMA), methacrylate pectin (PECMA), and methacrylate carboxymethyl cellulose (CMCMA) (Choi and Cha, 2019).

Photocrosslinkable sites of PECMA based bioink enabled to print chemically defined single-component 3D scaffolds with tunable mechanical strength (Pereira et al., 2018b). With increase in ink concentration from 1.5 wt% to 2.5 wt%, elastic modulus of hydrogel was adjusted from 79.6 Pa to 2,600 kPa under UV irradiation for 160 s (Pereira et al., 2018b). Moreover, extended UV exposure time from 160 s to 300 s, 3.2-fold increased stiffness modulus gels could be obtained in low polymer concentration (1.5 wt%) (Pereira et al., 2018b). In one study, methacryloyl-substituted tropoelastin (MeTro) and GelMA blend were photo-cured under UV (320–390 nm) light for 0.23 s per μm of thickness (Soucy et al., 2018). It showed 15 times higher adhesive strength than fibrin-based hydrogel with 85% encapsulated viable Schwann cells for 5 days (Soucy et al., 2018). It is expected to be used as a rapid tissue adhesive. In another research, HAMA/CMCMA hydrogel showed a much faster crosslink time of 0.018 s per μm thickness, under 400 nm visible light (Huang Y. C. et al., 2022). Based on its durable compressive elastic modulus (0.82 MPa), it is expected to be employed as an anti-adhesion barrier for post-operative measures (Huang Y. C. et al., 2022). By filling in the epidural defect space, the HAMA/CMCMA based scaffolds hinders the attachment and migration of 3T3 fibroblasts (Huang Y. C. et al., 2022). Thus, the fast polymerization time of methacrylate-based polymers has allowed them to be utilized as instantly and urgently needed biomaterials.

Especially, the intimate polymeric network between different types of biomaterials is the distinctive advantage of methacryloyl-mediated cross-linking. UV-induced dual crosslinked hydrogel consisting of methacryloyl-substituted Bletilla Striata polysaccharide and gelatin has suitable pore size (85.20 ± 4.99 µm), porosity (72.13% ± 2.15%) and significantly enhanced compression modulus (62.93 ± 8.24 kPa) compared to that of the single network which showed effective wound closure ability for diabetic wound treatment due to the tightly dual crosslinked network structure (Liu et al., 2022a).

Likewise, a wide range of spatially cross-linkable methacryloyl facilitates the fabrication of complex micro- or macro-structures as tissue engineering scaffolds using photo-patterning, bioprinting, and microfluidics (Hasany et al., 2021). Although both the biocompatibility and cytotoxicity of each methacryloyl-modified biomaterial should be identified for clinical applications, methacrylate crosslinked polymer is an ideal candidate for various tissue implants from the brain to bone now that it represents a wide range of elastic moduli (from mPa to GPa) (Chandrasekharan et al., 2019; Kono et al., 2020).

### 1.3 Benzophenone and diazirine

Diazirine and benzophenone are occurred photocrosslinking related with redox and generate intermediates during the reaction process (Wu and Kohler, 2019). The intermediates, which are comprised of covalent bond have greatly reactive crosslinking activity and can interaction with various ligand of proteins or residue of polymers (Deseke et al., 1998).

**FIGURE 4:**
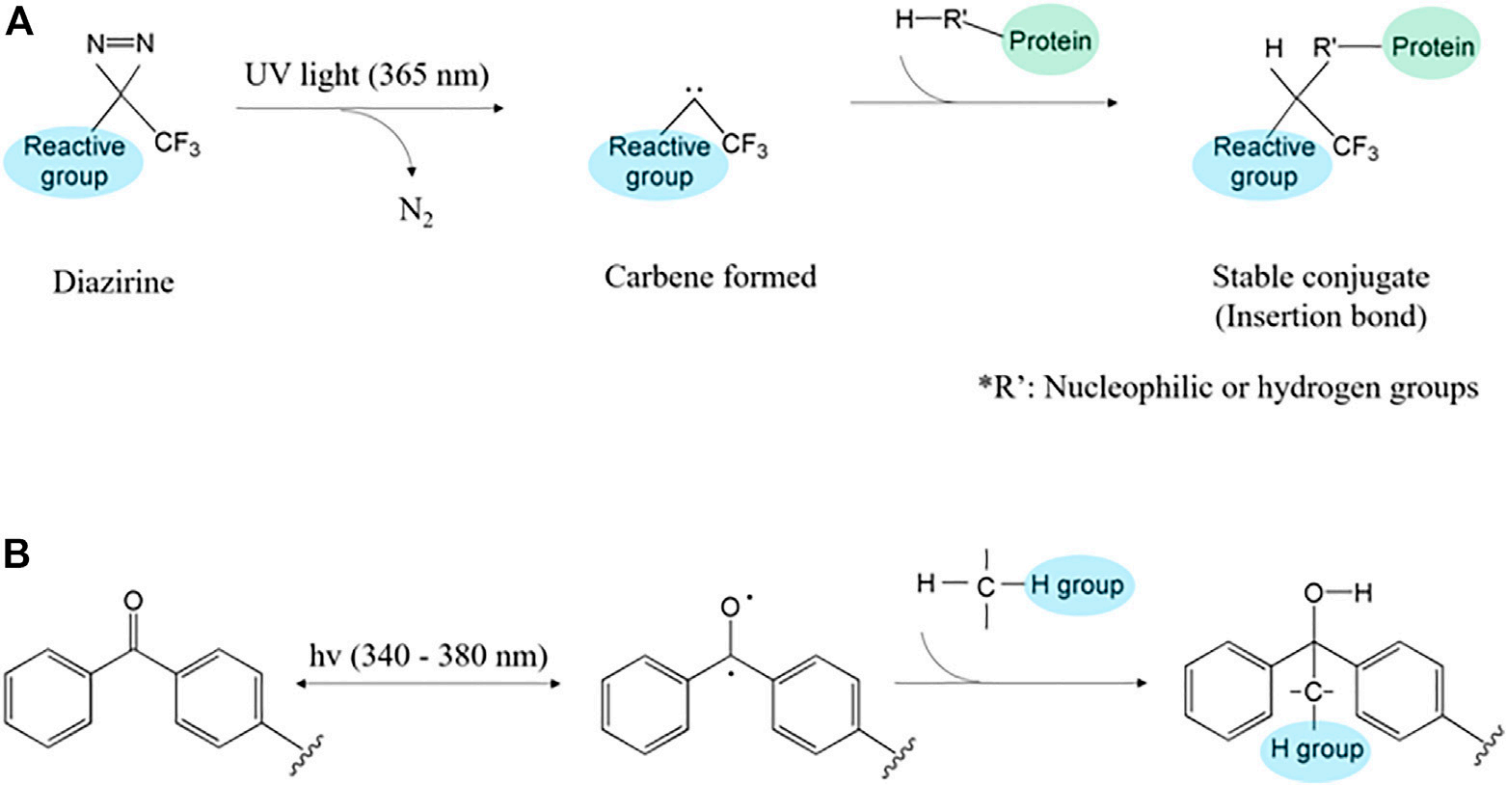
The protein polymerization mechanism through photocrosslinking of **(A)** diazirine and **(B)** benzophenone.

Specifically, the diazirine has aromatic azides structure (Kuboe et al., 2010). It comprises one carbon and two nitrogen atoms connected with a double bond, forming a cyclopropene-like ring (Zhou et al., 2020). The carbene in diazirine rapidly binds with other biomolecules through C-H, O-H, and N-H bonds under UV (350–380 nm) irradiation (Hill and Robertson, 2018; Musolino et al., 2021). Specifically, when the UV light is irradiated to diazirine, N_2_ gas is extruded and forms singlet carbene (Dey et al., 2021). The singlet carbene combines with nearby biomolecules through covalent, C-H, and heteroatom-H bonds, thus forming isomerized carbene (Figure 4A) (Murale et al., 2017; West et al., 2021; Yu and Baskin, 2022).

This reactivity is not constrained to light only, but it also occurs due to heat and electricity (Álvarez-Hernández et al., 2018; Lepage et al., 2019). Although the diazirine has high thermal and chemical stability, the process of photocrosslinking reaction is more complex than benzophenone (Ge et al., 2018; Jakubovska et al., 2018). However, the carbene, which is intermediate material during reaction, is not uneconomical because carbene can be utilized for crosslinking (Temel et al., 2011). The carbene and diazirinyl radicals have been exploited for bulk polymer crosslinking, molecular labeling, and target identification and dazirine has been extensively exploited for surface modification of biomaterials (Ye et al., 2018).

The diazirine is applied to the conjugation and reinforces physical strength. In the study related to the conjugation, the diazirine conjugated GFOGER peptide showed enhanced cell (HT1080) adhesion and spreading when mixed with film (Malcor and Mallein-Gerin, 2022). And the diazirine conjugated elastic-like protein is formed a carbene intermediate and can be inserted rapidly into the located close conjugated protein releasing N_2_ (Raphel et al., 2012). The conjugated protein is made into a film that has 50 μm thickness, is sturdy enough to withstand a weight of 4.5 g, and can maintain the cell metabolism system (6 days) (Raphel et al., 2012). The rapid (60 s) gelation of diazirine led to an oral adhesive application that involved the chemical curing of bromo-diazirine to form carbene of bacterial cellulose (Singh et al., 2021). The rapid gelation (within 60 s) promoted low flowability with up to 35 kPa adhesiveness in wet conditions, which is suitable for mucoadhesive drug delivery (Singh et al., 2021).

Benzophenone, which consists of the carbonyl group and two benzene groups, forms two radicals when activated by ultraviolet (350–365 nm) light (Anderson and Castle, 2003; Neumann et al., 2013; Fu and Chang, 2019; Sharma et al., 2021; Xue et al., 2021). Benzophenone shapes triplet-state ketone and forms C-C bonding (Liu et al., 2020; Olson et al., 2020). Then, the formed radicals react with the neighboring C-H bond**,** extract protons of the carbon chain, and create methyl-radical at the surface (Neumann et al., 2013). The formed radical reacts with hydrogen bonds such as carbon, nitrogen, or oxygen (Figure 4B) (Yagci et al., 2010; Lago et al., 2015; Fu and Chang, 2019). The triplet-state, which is formed during the reactive process, is related to electron donation and diradical (Porter and Suppan, 1965). Thus, various solutions are involved in different contents of atoms and it can regulate bonding by using different solutions (Christensen et al., 2012). It unnecessary co-initiator because they performed photosensitizer and hydrogen donor (Temel et al., 2011). Additionally, its non-polar property allows benzophenone to be stable underwater (Ge et al., 2018). The tolerance for oxygen and water promotes its applicability in printing and coating materials (e.g., sunglasses coating and plastic colorings) (Keskin et al., 2018; Wang et al., 2018).

Benzophenone is a reinforcement or UV-supported agent (Yuk et al., 2016). The photo-crosslinking has been employed with chitosan/polyethylene oxide (PEO) fibers to transform PEO into a water-insoluble material (Kianfar et al., 2019). As a result, this enhances the solubility resistance (water or organic solvents) and thermal stability (Wang et al., 2018). The benzophenone introduction also helps the carboxyl group of the cellulose nanofibrils (CNF) to increase the tensile strength of the CNF gel, even in wet conditions (Orelma et al., 2016). Experimentally, the cured CNF gel (138 MPa cm^3^ g^-1^) showed high tensile strength compared with non-cured CNF gel (109 MPa cm^3^ g^-1^) (Orelma et al., 2016). Furthermore, the hydrogel-elastomer hybrid (polyacrylamide (PAAm)-alginate, PAAm-hyaluronan, PAAm-chitosan, polyethylene glycol diacrylate (PEGDA)-alginate and PEGDA-hyaluronan) can have minute structure and interface toughness increase due to benzophenone (200 to 900 Jm^-2^) (Yuk et al., 2016). Collagen-GAG biomaterial platform can regulate the mechanical properties independently by curing with benzophenone and identify metabolizing activity, proliferation, and gene expression of the adipose-derived mesenchymal stem cells (ASCs) based on mechanical properties (Banks et al., 2014). Also, collagen treated modified benzophenone (benzophenone dimer) can make sub-micrometer or micrometer scaffold through multiphoton excited photochemistry (Basu et al., 2005).

Owing to benzophenone and diazirine using a UV light for crosslinking, the cells are significantly less negatively affected corresponding UV range (350–380 nm) (Anderson and Castle, 2003). So, they are not caused the harmful effect to cells and not damage biological (Chou et al., 2011; Hill and Robertson, 2018).

### 1.4 Cinnamoyl

**FIGURE 5:**
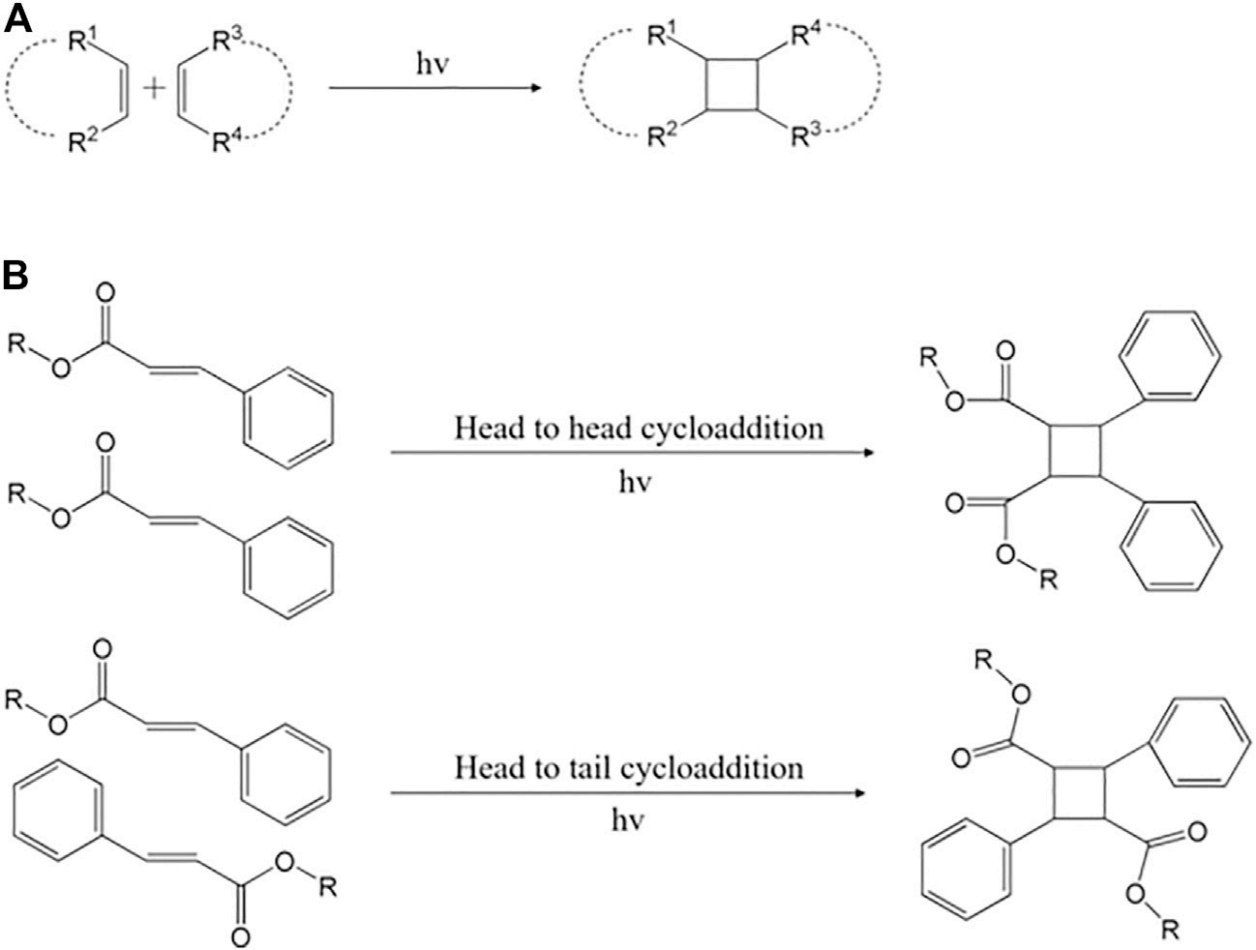
Brief mechanism of **(A)** cyclobutane and **(B)** (2 + 2) photocycloaddition (Gupta et al., 2004; Sarkar et al., 2020).

The carboxy group deprotonation of cinnamic acid generates UV-sensitive cinnamate groups (Shi et al., 2009; Bobula et al., 2015; Zhang et al., 2020; Gadek et al., 2021). Those cinnamates are photo-dimerized through (2 + 2) photocycloaddition (Figure 5A). The photocrosslinking of cinnamate has a unique characteristic of reversible reactions. Photo-dimerization is dominant longer than 260 nm, whereas photocleavage happens less than 260 nm (Nakayama and Matsuda, 1992). It means that the formation and division of the photo-crosslinking can be adjusted by the wavelength range. Using this, latex-based sizing nanoparticles react reversibly to wavelengths, suggesting the emergence of new carriers in environmental and biomedical fields (Shi et al., 2008). It also manufactured a hydrogel film treated with cinnamoyl on the surface to prove its ability to heal itself using photoreaction (Froimowicz et al., 2011). In addition, the self-healing ability of bio-based lignin and glycerol-derived monomers with cinnamate groups was optimized to help design a new photocrosslinking-based self-healing product that responds environmentally friendly and reversibly (Sinha Roy et al., 2021).

In addition, cinnamate forms (2 + 2) cyclobutane and is attracting great attention because it can perform photo-crosslinking without a photoinitiator (Bobula et al., 2015; Schelkle et al., 2016). The irritation induces two olefins of the alkenyl group in the cinnamate and forms a cyclobutene capable of photo-crosslink (Gupta et al., 2004). During the cycloaddition process, enone is bonded with olefin. At this time, the negative end of the alkene group dipole with olefin and the β carbon of enone are combined (Figure 5B) (Corey et al., 1964). The β-alkenyl-substituted enone has a steric hindrance that determines the region and stereoselectivity of the polymer (Sarkar et al., 2020).

Since the olefin structure is present in the side chain, photo-crosslinking of the cinnamate is limited to the side chain polymer (Schelkle et al., 2016). However, (2 + 2) photo-reduction addition reactions are considered an efficient synthesis method because the molecular binding is fast and predictable (Hoffmann, 2008; Sanyal, 2010; Cardenas-Daw et al., 2012), especially because they occur easily in cinnamoyl (Rennert et al., 1967; Schelkle et al., 2016).

Using a series of advantages, cinnamic acid is often used in various ways for natural polymer synthesis. It is also welcomed in the photo-activated polymer (Balaji et al., 2003) and patterned polymer (Kim et al., 2009) areas because it exhibits stable reactivity without a photoinitiator and photosensitizer (Fertier et al., 2013).

Hyaluronan and trans-cinnamic acid were employed as photocurable derivatives to generate a water-insoluble microfiber (Bobula et al., 2015). The anhydride of cinnamic acid reacted with hyaluronan to be acylated (Bobula et al., 2015). As a similar manner, gellan gum films are negatively charged, enabling long-term electrostatic repulsion of bacteria (Lee et al., 2012). In addition to these, photocrosslinking was carried out with chitosan (Wu et al., 2007) and starch (Zhang et al., 2020), and physical or mechanical properties were improved to alleviate low durability problems. Octanoyl chitosan cinnamate synthesized using a regioselective variant of chitosan have been shown to form a stable monolayer by dispersing at the interface between air and water (Wu et al., 2007). The backbone of OCC maintained chirality in the film and facilitated optical characterization (Wu et al., 2007). Cinnamic acid-modified starch (CA-St) was used for the nanoprecipitation and photo-crosslinking of the colloidal particles to fabricate colloidal particles (CPs) (Zhang et al., 2020). The cycloaddition of cinnamic acid-modified starch CPs alleviates low durability problems in intravenous administration or drug loading/release (Zhang et al., 2020). Moreover, the hydrophobic moieties allow the starch to deliver relatively hydrophobic drug molecules (Zhang et al., 2020). Cinnamated-collagen made using EDC/NHS conjugating decreased storage modulus as the cinnamate content increased (Dong et al., 2005). Scaffold was produced using hydrogels using gelatin one-pot synthesis, which can be used appropriately for biomaterial applications because various trigger reactions exist and are not cytotoxic to fibroblasts (Gattás-Asfura et al., 2005).

Also, it was confirmed that the physical properties were improved by completing crosslinking within 60 min in both hydrated gel and dry film, and the potential applicability of collagen-based materials in drug delivery and tissue engineering was improved (Dong et al., 2005).

### 1.5 Norbornene

**FIGURE 6:**
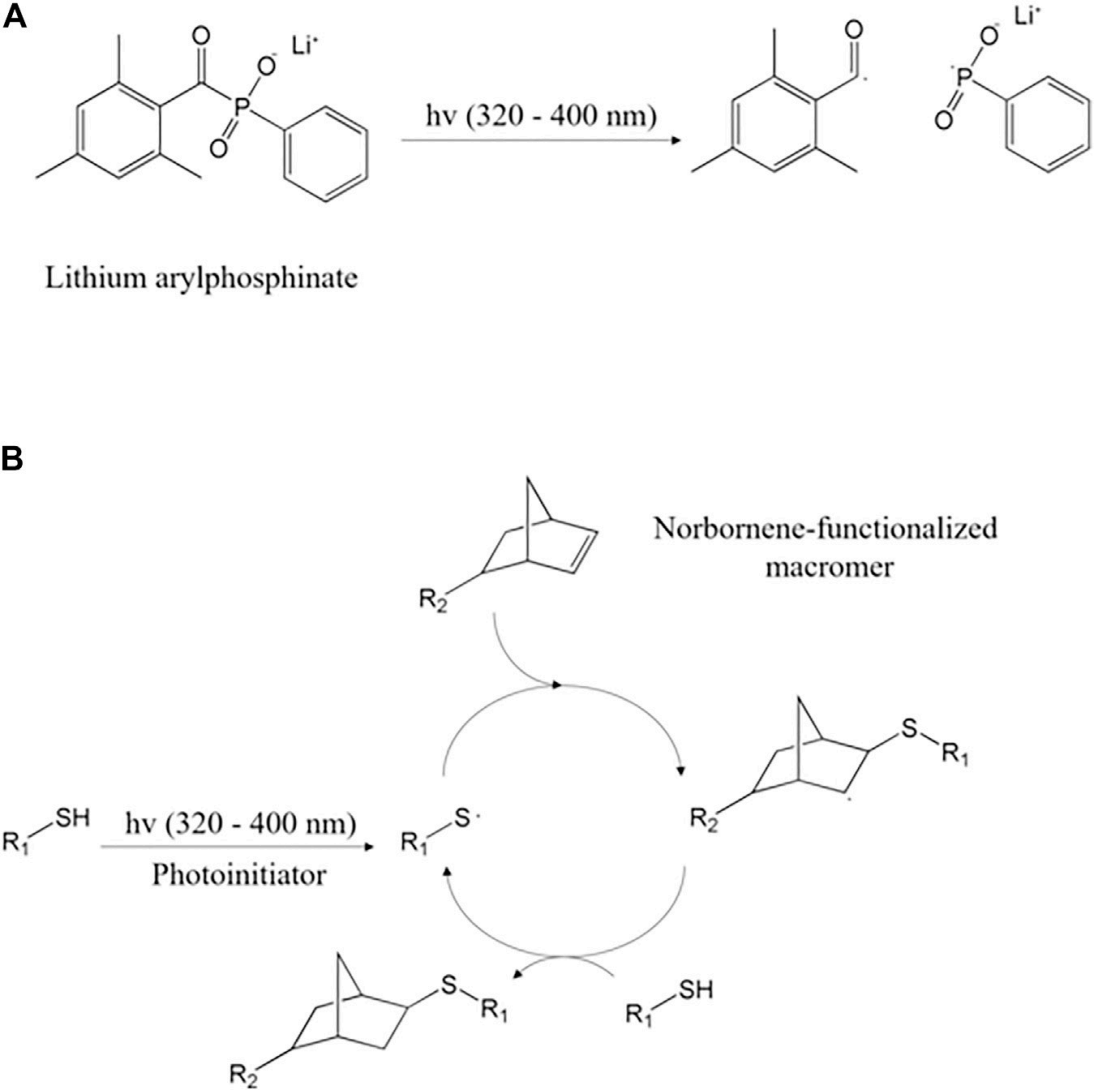
**(A)** LAP decomposition by UV and **(B)** thiol–norbornene photo-click reaction step with a thiol–containing molecule (R1–SH) (Lin et al., 2015).

Norbornene is a cyclic alkene mainly used as a monomer and an intermediate for organic synthesis and is a particularly reactive ene compared to alkene due to the deformation of the inherent ring (Blasco et al., 2017). Norbornene moiety reacts with thiolated agents and forms crosslinkage, under UV light. Thiols react with various ene functional groups such as alkene, vinyl ether, and acrylate. Among them, however, norbornene moiety is commercially valuable because it is most reactive and progresses quickly (Hoyle and Bowman, 2010; Blasco et al., 2017; Nguyen et al., 2021). Norbornene is incorporated into natural polymers through amide reactions (Devaraj et al., 2008). Natural polymers incorporated with norbornene exhibit low solubility under acidic aqueous solvent conditions (Mũnoz et al., 2014; Alves et al., 2022). This is because the overall hydrophobicity was increased by the norbornene moiety, and the positive elective charge was reduced due to the conversion of the amine into the amide (Michel et al., 2019). In general, norbornene is incorporated into a natural polymer in the presence of carboxy anhydride (Devaraj et al., 2008). It has the advantage of improving the solubility of the natural polymer in the aqueous medium than before and is very stable *in vivo* (Devaraj et al., 2008; Michel et al., 2019). The mechanism of this thiol-norbornene photo-crosslinking reaction is initiated by a type I photoinitiator (typically LAP or Irgacure 2959), which is photo-decomposed into radicals by UV (UV-A, 320–400 nm) light (Figure 6A) (Hoorick et al., 2020). The decomposed photo-initiator radicals extract hydrogen atoms from the thiol of the thiol-containing molecule (R1-SH) and produce thiyl radicals (Lin et al., 2015). The produced thiyl radical crosses the norbornene carbon-carbon double bond of the norbornene-functionalized macromer (R2-norbornene), subsequently producing norbornene radical (Lin et al., 2015). Norbornene radical extracts hydrogen atoms from thiol complete thioether bonds, and regenerates thiyl radical (Figure 6B) (Cramer et al., 2003; Lin et al., 2015). This photo-crosslinking reaction proceeds at a stoichiometric rate until thiol or norbornene is depleted and is crosslinked step by step (Lin et al., 2015). Norbornene’s high reactivity to thiol radical and low reactivity to norbornene radical alleviates the non-specific photo-crosslinking (Gramlich et al., 2013).

In rare cases, thiol-norbornene photo-crosslinking by visible light can occur, similar to the mechanism of UV-based photo-crosslinking systems, except that type II photoinitiator (non-cleavage-type) (e.g., eosin-Y, rose bengal) is used (Lin et al., 2015). Thiol-norbornene has been utilized as a photo-crosslinking moiety of natural biopolymer materials, including chitosan, gelatin, collagen, and pectin, especially for tissue engineering applications (Mũnoz et al., 2014; Pereira et al., 2018a; Michel et al., 2019; Guo et al., 2021).

As for chitosan, the norbornene moiety is acquired by amide bond formation, which is initiated by the reaction between chitosan and carbic anhydride [norbornene-derived chitosan (CS-nbn-COOH)] (Michel et al., 2019, 2020). In one research, thiolated diethylene glycol (HS-DEG-SH) was used as a crosslinker and Irgacure 2959 was utilized as a photoinitiator [UV-A (320–400 nm)] (Michel et al., 2019). The UV-A exposure time was between 20 s and 30 min (Michel et al., 2019). Similar procedures are applied to other natural polymers. For instance, in another study, the primary amine of gelatin reacted with carbic anhydride at 50°C for 2 h to form norbornene through amide bonding (Mũnoz et al., 2014). LAP was used as a photoinitiator and dithiothreitol (DTT) was added as a crosslinker (Guo et al., 2021). Pectin required VA-086 as a photoinitiator and dimethyl sulfoxide (DMSO) as a crosslinker in a different study (Pereira et al., 2018a). The manufactured hydrogel is a scaffold in tissue engineering and plays a role in skin wound healing, 3D printing, and tissue environment formation (Sun et al., 2011). Methacrylated collagen and GelMA hydrogel formation with random chain growth photopolymerization are unstable and yield high concentrations of initial radicals (Brinkman et al., 2003; Gaudet and Shreiber, 2012; Guo et al., 2021). Therefore, it is not ideal for cell-containing hydrogel formation and is rarely used in bioprinting (Lin et al., 2011; Mũnoz et al., 2014; Guo et al., 2021). Hydrogels formed from thiol-norbornene are suitable for cell-containing hydrogel formation in this respect because they can be photo-crosslinked at low radical concentrations with very fast reactions and induce cell proliferation (Guo et al., 2021). In particular, NorCol (norbornene-functionalized collage)/gelatin bio-ink showed good printability by controlling light and temperature at once, and cells showed great potential by showing excellent viability within bio-printed hydrogels (Guo et al., 2021). In addition, it is important to establish a pertinent *in vitro* culture system for liver cells to understand the mechanism of liver disease progression or recovery (Lau et al., 2012; Greene and Lin, 2015). At this time, norbornene-functionalized gelatin (GelNB) hydrogels can be used as a three-dimensional scaffold capable of designing parts of the cell-extracellular matrix (ECM) interactions important for cell survival and function (Lau et al., 2011; McCall and Anseth, 2012; Greene and Lin, 2015).

### 1.6 Aryl azide

Aryl azide is a moiety containing azide groups (-N3) as a functional group or substituent derived from aromatic hydrocarbons. Aryl azide is mainly made by replacing diazonium salts or aryl halide with sodium azide (Liu and Tor, 2003). When exposed to light sources, aryl azide releases nitrogen molecules to produce electron-deficient nitrene that can be inserted into the C-H bond (Ono et al., 2000; Liu and Tor, 2003). It facilitates covalent bond formation between natural biopolymers (Zhu and Ma, 2004). The hydrogel is generated by UV irradiation, and the increase in its exposure time results in better mechanical properties and lower swelling ratio (Cho et al., 2016).

**FIGURE 7:**
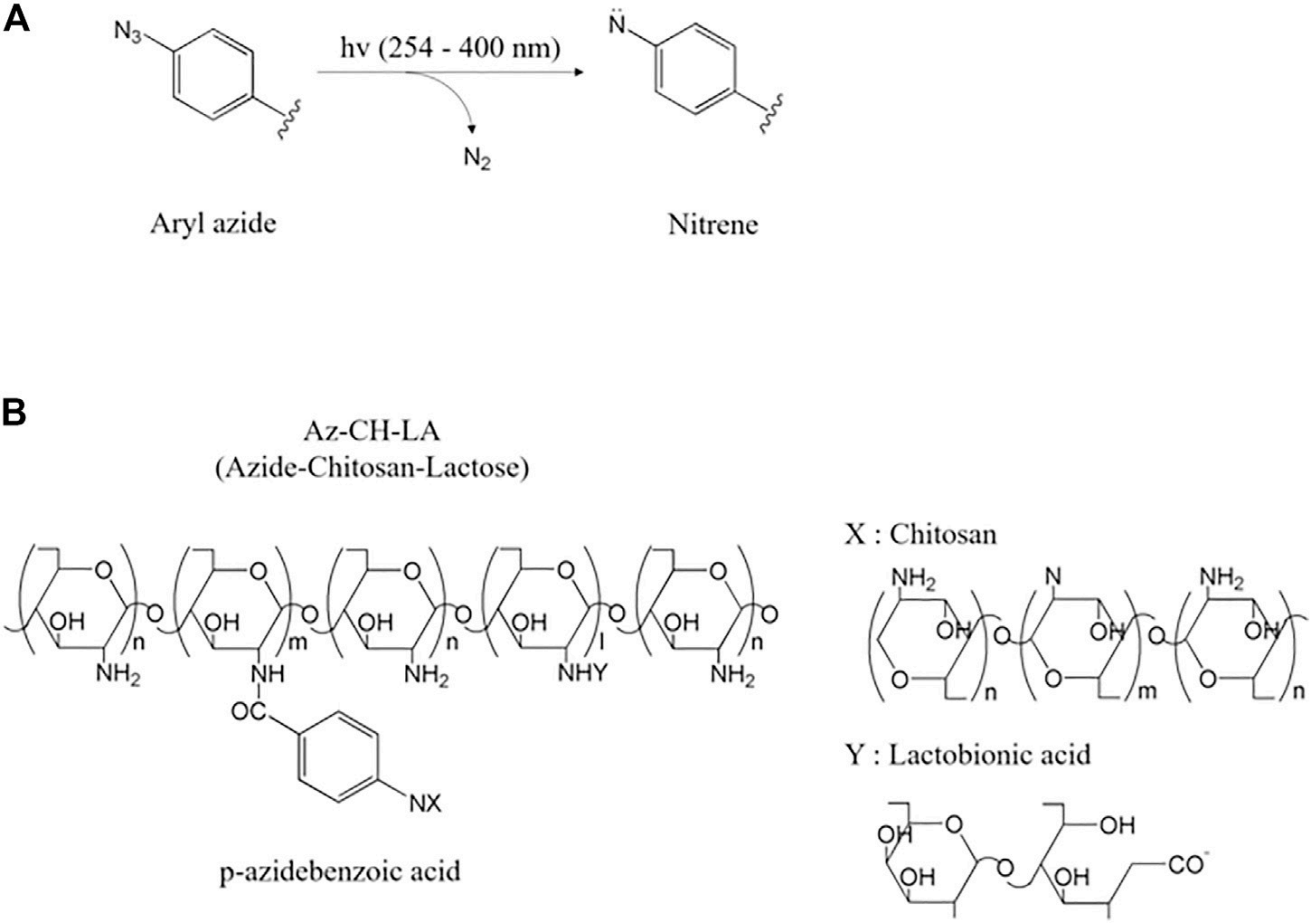
**(A)** Aryl azide moiety is converted into highly reactive nitrene by releasing N₂ under UV light (Tanaka et al., 2008) and **(B)** azide-chitosan-lactose (Az-CH-LA) obtained by photo-crosslinking (Az-CH) and dehydration condensation reaction (CH-LA) (Ono et al., 2001; Tanaka et al., 2008).

The mechanism of the aryl azide photo-crosslinking reaction starts with UV irradiation. The azide group (-N3) releases nitrogen molecules (N2) and is converted into highly reactive nitrene groups (Ono et al., 2000). Nitrene groups with two unshared electron pairs interact very quickly with the amino groups of the natural biopolymer to be bonded to form azo groups (-N=N-) and become crosslinked (Figure 7A) (Ono et al., 2000). Through this photo-crosslinking process, gelation is completed. If the proper reaction site is not nearby, nitrene is rearranged into the more stable ketenimine and has a disadvantage in that crosslinking efficiency is lost (Tanaka et al., 2008). The maximum absorption wavelength of azide is 250 nm, but the absorption amount is increased through substitution with aryl azide; thus, a wavelength of over 400 nm can be used (Ono et al., 2000; Tanaka et al., 2008). This wide-range reactivity allows the aryl azide compound to react with various biomolecules (Tanaka et al., 2008).

In one study, this crosslinking method was employed with an azide (p-azidebenzoic acid) conjugated chitosan (Ono et al., 2000). The rapid gelation [60 s, under UV (254 nm) light and Irgacure 2959] and dense network retarded the encapsulated drug release (Figure 7B) (Ono et al., 2000; Sydow et al., 2019). In tissue engineering, these photochemical biopolymers are used as scaffolds for potential drug and cell delivery to tissues and biological activity is enhanced by photo-crosslinking (Sydow et al., 2019).

The properties of photo-crosslinking can be used to detect protein-protein interaction (PPI) in living cells using the aryl azide moiety as a probe (Baruah et al., 2008; Pham et al., 2013; Mishra et al., 2020). These aryl azide ligases are incorporated into proteins and when irradiated with UV (300–360 nm), produce reactive species that interact with other proteins nearby, forming covalent bonds (Baruah et al., 2008; Mishra et al., 2020). It has advantages over other PPI detection methods in that it can detect endogenous protein interactions (Baruah et al., 2008; Mishra et al., 2020).

In addition, medical tissue adhesives use reactive groups to produce shared crosslinking, which includes aryl azide that exhibit spontaneous and highly reactive crosslinking as reactive groups (Ono et al., 2000; Nam and Mooney, 2021). Therefore, aryl azide photo-crosslinking hydrogel using UV is mainly used as a tissue adhesive with cell compatibility (Rickett et al., 2011; Nam and Mooney, 2021).

### 1.7 Triethanolamine

Triethanolamine (TEA) is a sacrificial electron donor involved in the photopolymerization reaction of eosin (Popielarz and Vogt, 2008; Wong et al., 2015). The red-colored eosin is a photocatalyst (photoinitiator) and generates radicals at a reactive moiety of natural polymers such as cellulose, gelatin, and chitosan (Lim et al., 2008; Haria and König, 2014). Photocrosslinking using radicals has the high stability of a shared crosslinked network and gelates the surroundings quickly (Shih and Lin, 2013).

**FIGURE 8:**
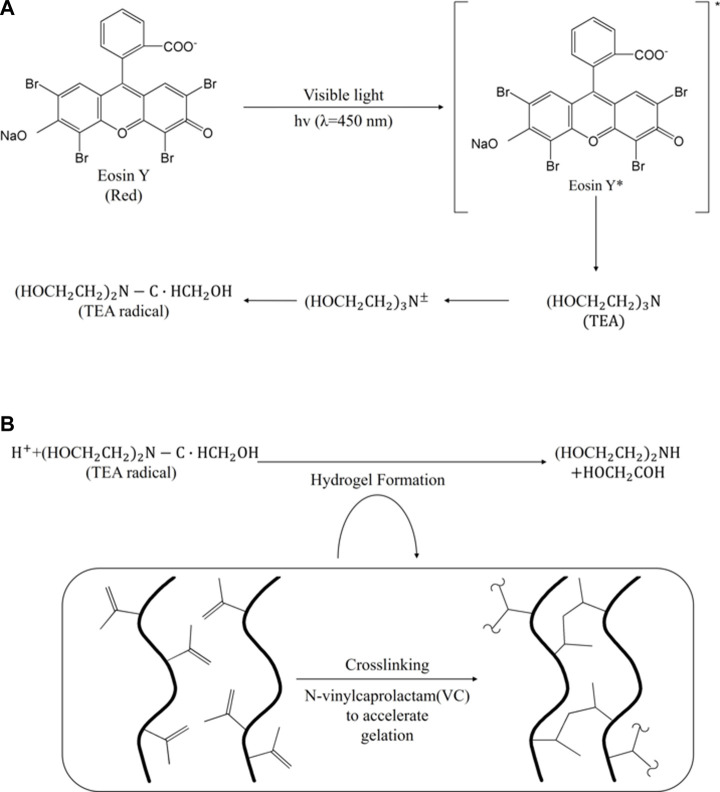
The mechanism of **(A)** eosin-based photopolymerization and its **(B)** hydrogel formation (Noshadi et al., 2017).

The TEA transfers electrons to excited eosin, producing anionic and triethanolamine cation radicals once the eosin (eosin Y) is excited in a triplet state under visible light (400 nm < *θ* < 700 nm) (Noshadi et al., 2017). Subsequently, it leads to rapid proton loss from the triethanolamine radical cation (TEA·+) to neutral R-amino radical (TEA·) (Noshadi et al., 2017). The protons are then transferred to eosin anionic radicals to produce neutral eosin radicals (Figure 8) (Valdes-Aguilera et al., 1992; Noshadi et al., 2017). TEA radicals initiate polymerization with monomers with vinyl groups, such as poly (ethylene glycol) diacrylate (PEG-diacrylate) and vinylpyrrolidone (VP), and are also used to accelerate gelation of covalent monomers (e.g., N-vinylpyrrolidone or NVP) (Elbert and Hubbell, 2001; Popielarz and Vogt, 2008). A 3D -printed isomalt structure made of a carbohydrate glass material was coated through poly (ethylene glycol) diacrylate (PEGDA) and surface-initiated photopolymerization (Chen et al., 2020). Coating with Eosin/TEA photopolymerization had the advantage of isomalt miscibility and stability at high temperatures and showed the potential to create physiologically related tissues by facilitating the construction of biomimetic vascular structures in various hydrogels (Chen et al., 2020).

The Eosin Y-TEA reaction is co-initiator and co-monomers dependent, and the use of high TEA concentrations can cause undesirable cytotoxic effects on some sensitive cell types, so caution should be taken (Hao et al., 2014). Nevertheless, stability and safety are evaluated to prevail over the use of ultraviolet initiators because visible light initiators can reduce the risk to proteins and DNA (Avens et al., 2008). Gelatin-methacrylate (GelMA) bioink, whose viscosity was adjusted using silk fibroin particles, improved cell suspension and proved to be a biocompatible material through non-cytotoxic and high level of metabolic activity (Na et al., 2018). Since hydrogels cured under visible light showed stability for a long time (18 months) from exfoliation compared to ultraviolet (UV)-photocured hydrogels, visible light-induced photocrosslinking is promising to be applied to the 2D patterning of hydrogels (Kizilel et al., 2004). In PEGDA scaffolds consisting of 1-vinyl-2-pyrrolidone, we define non-toxic conditions for photoencapsulation of human mesenchymal stem cells, enhancing the viability of human mesenchymal stem cells and generating hydrogel scaffolds with tightly bridged networks (Bahney et al., 2011). Through this, it can be confirmed that it is desirable to apply it to biological applications such as cell encapsulation (Avens et al., 2008; Occhetta et al., 2015).

Due to these advantages, Eosin–coinitiator photocrosslinking has been used for various purposes such as encapsulation, drug testing, and biosensing in addition to bioink formulations for 3D bioprinters. *In situ* photocroslinkable hyaluronan, which encapsulated Chondrocytes, promoted the maintenance of cartilage phenotype and cartilage matrix synthesis, accumulated a significant amount of cartilage matrix, and was evaluated as a scaffold for repairing cartilage by accelerating healing *in vivo* osteochondral defects (Nettles et al., 2004). Photopolymerizable Dock-and-Lock hydrogel, which can be used as a scaffold to support fast self-assembled cells and drugs, can control moduli according to the duration of light exposure and can be used for various purposes depending on the unique physical properties of the gel (Lu et al., 2013) Visible ray-induced gelation of methacrylate materials (gelatin methacrylate (Gel-MA) and methacrylate alginate (Alg-MA)) showed high potential for surgical tissue sealing for *in vivo* systems (Charron et al., 2016; Kumar et al., 2022).

## 2 Conclusion

This review covered photo-reactive moieties and their mechanisms. Their application examples in tissue engineering were also dealt with. The whole part was classified into free-radical chain polymerization (tyrosine, tyramine, methacrylol, cinnamoyl, and eosin-based photopolymerization), thiol-ene photo-crosslinking (norbornene), and photo-mediated redox crosslinking (benzophenone, aryl azide, and diazirine) (Table 1) (Lim et al., 2020a; Lim et al., 2020b).

**TABLE 1:** Summary of photo-reactive moieties used in tissue engineering.

Moieties	Name of initiator	Irradiation source (nm)		Characteristics	Natural polymers	Applications in tissue engineering	References
**Tyrosine and tyramine**	2-hydroxy-1-[4-(2-hydroxyethoxy) phenyl]-2-methyl-1-propanone (Irgacure 2959)	UV	365	Free-radical- chain polymerization, Ru (Ⅱ) polypyridine/persulfate method	Silk fibroin, keratin, fibrinogen, marine-derived aneroin protein, recombinant resilin, collagen, tyrosine-rich peptides	Elastic gel as ocular prostheses, alginate-tyramine microfibers, natural polymer-based inks, hydrogels as cartilage-binding glue	Liu et al. (2021); Raven et al. (1971); Gillespie. (1972); Fancy and Kodadek. (1999); Elvin et al. (2009); Sando et al. (2010); Truong et al. (2011); Houée-Lévin et al. (2015); Min et al. (2016); Partlow et al. (2016); Navarro et al. (2018); Choi and Cha. (2019); Park et al. (2019); Lee et al. (2019); Mu et al. (2020); Gong et al. (2020); Lim et al. (2020a); Liu et al. (2021); Perin et al. (2022); Huang et al. (2022b)
	tris (2,20-bipyridyl) dichlororuthenium (II) (Ru(II))/persulfate	visible	450, 452				
**Methacrylol**	2-hydroxy-1-[4-(2-hydroxyethoxy) phenyl]-2-methyl-1-propanone (Irgacure 2959), lithium phenyl-2,4,6-trimethylbenzoylphosphinate (LAP)	UV	365–405	Free-radical- chain polymerization	Silk fibroin, gelatin, hyaluronic acid, chitosan, alginate, starch, pectin	adhesive hydrogel for nerve regeneration free from microsurgical suturing, hydrogel for prevention of dural defects, bio-ink	Messager et al. (2013); Noshadi et al. (2017); Pereira et al. (2018b); Soucy et al. (2018); Basara et al. (2019); Han et al. (2020); Lim et al. (2020a); Noè et al. (2020); He et al. (2021); Kim et al. (2021c); Huang et al. (2022a)
	tris (2,20-bipyridyl) dichlororuthenium (II) (Ru(II))/persulfate, eosin Y	visible	420–450				
			450–550				
**Benzophenone and diazirine**	Type II free radical photoinitiator	UV	350–380, 365	Free-radical-chain polymerization	Chitosan, collagen, silk fibroin	Benzophenone-modified dextran-based hydrogel for bone regeneration and implant fields, drug delivery, photo-affinity labeling field in protein as a probe	Neumann et al. (2013); Anderson and Castle. (2003); Neumann et al. (2013); Ye et al. (2018); Hill and Robertson. (2018); Keskin et al. (2018); Wang et al. (2018); Fu and Chang. (2019); Dey et al. (2021); Xue et al. (2021); Musolino et al. (2021); Sharma et al. (2021); Singh et al. (2021)
**Cinnamoyl**	N/A	UV	260	[2 + 2] cycloaddition reaction	Hyaluronic acid (HYA), starch, gellan gum	Microfiber, film, and colloidal particles (CP) for medical use	Gupta et al. (2004); Shi et al. (2009); Bobula et al. (2015); Zhang et al. (2020); Gadek et al. (2021); Sinha Roy et al. (2021)
**Norbornene**	2-hydroxy-1-[4-(2-hydroxyethoxy) phenyl]-2-methyl-1-propanone (Irgacure 2959), lithium phenyl-2,4,6-trimethylbenzoylphosphinate (LAP), 2,2′-Azobis [2-methyl-N-(2-hydroxyethyl) propionamide] (VA-086)	UV	320–400	Free-radical- chain polymerization	Chitosan, gelatin, collagen, pectin	Microgel for biopharmaceutical delivery, hydrogel as bio-ink and for skin wound healing and tissue environment formation	Hoorick et al. (2020); Yagci et al. (2010); Mũnoz et al. (2014); Lago et al. (2015); Lin et al. (2015); Pereira et al. (2018a); Fu and Chang. (2019); Michel et al. (2019); Hoorick et al. (2020); Guo et al. (2021)
	type II (e.g., eosin-Y, rose bengal)	visible	—				
**Aryl azide**	2-hydroxy-1-[4-(2-hydroxyethoxy) phenyl]-2-methyl-1-propanone (Irgacure 2959)	UV	250–400	Free-radical-chain polymerization	Chitosan	Hydrogel as a tissue adhesive, nanoparticles as drug delivery system	Ono et al. (2000); Liu and Tor. (2003); Tanaka et al. (2008); Cho et al. (2016); Sydow et al. (2019)
**Triethanolamine**	Eosin-Y and co-initiator	visible	450–550	Free-radical- chain polymerization	Gelatin, alginate, fucoidan	Hydrogel as bio-ink for 3D bioprinter, surgical tissue sealing for *in vivo* systems, cell culture matrix, and drug delivery	Lim et al. (2008); Cruise et al. (1998); Lim et al. (2008); Haria and König. (2014); Charron et al. (2016); Kumar et al. (2022)

Since photo-crosslinking has unique and unparalleled merits in specific controllability and invasiveness, many moieties have been chemically incorporated into natural polymers to facilitate its photo-reactivity, as well as generate covalent networks. Especially, this photo-initiated crosslinking was welcomed by natural polymers due to their poor mechanical properties which deterred their wide applications (Sundaramurthi et al., 2014; Choi and Cha, 2019). Employing a variety of photocrosslinking moieties regulate not only mechanical, but also biological, and physicochemical properties with a wide range (Zhao et al., 2013).

The tissue matrixes that have variety mechanical properties through regulating the photocrosslinking affect cellular behavior like cell growth, differentiation, and proliferation (Camp et al., 2020). Thus, it can develop the scaffold that have variety mechanical properties and can biodegradable completely using union such as protein-protein binding, hydrogel-based polysaccharide, and gene binding. Moreover, photocrosslinking can applicate not only scaffolds that have various mechanical properties range such as bone, cartilage, tissue, and organ but also biocompatible drug delivery, biosensor, and bio-probes. Likewise, the photocrosslinking technique is promising technology for tissue engineering fields.

## References

[B1] Álvarez-Hernández M. H. Artés-Hernández F. Ávalos-Belmontes F. Castillo-Campohermoso M. A. Contreras-Esquivel J. C. Ventura-Sobrevilla J. M. (2018). Current scenario of adsorbent materials used in ethylene scavenging systems to extend fruit and vegetable postharvest life. Food Bioprocess Technol. 11, 511–525. 10.1007/s11947-018-2076-7

[B2] Alves P. M. Pereira R. F. Costa B. Tassi N. Teixeira C. Leiro V. (2022). Thiol-norbornene photoclick chemistry for grafting antimicrobial peptides onto chitosan to create antibacterial biomaterials. ACS Appl. Polym. Mat. 4, 5012–5026. 10.1021/acsapm.2c00563

[B3] Anderson W. A. C. Castle L. (2003). Benzophenone in cartonboard packaging materials and the factors that influence its migration into food. Food Addit. Contam. 20, 607–618. 10.1080/0265203031000109486 12881135

[B4] Applegate M. B. Partlow B. P. Coburn J. Marelli B. Pirie C. Pineda R. (2016). Photocrosslinking of silk fibroin using riboflavin for ocular prostheses. Adv. Mat. 28, 2417–2420. 10.1002/adma.201504527 26821561

[B5] Avens H. J. Randle T. J. Bowman C. N. (2008). Polymerization behavior and polymer properties of eosin-mediated surface modification reactions. Polym. Guildf. 49, 4762–4768. 10.1016/j.polymer.2008.08.054 PMC2614886 19838291

[B6] Bahney C. S. Lujan T. J. Hsu C. W. Bottlang M. West J. L. Johnstone B. (2011). Visible light photoinitiation of mesenchymal stem cell-laden bioresponsive hydrogels. Eur. Cell. Mat. 22, 43–55. 10.22203/ecm.v022a04 PMC5050040 21761391

[B7] Balaji R. Grande D. Nanjundan S. (2003). Studies on photocrosslinkable polymers having bromo-substituted pendant cinnamoyl group. React. Funct. Polym. 56, 45–57. 10.1016/s1381-5148(03)00031-2

[B8] Banks J. M. Mozdzen L. C. Harley B. A. C. Bailey R. C. (2014). The combined effects of matrix stiffness and growth factor immobilization on the bioactivity and differentiation capabilities of adipose-derived stem cells. Biomaterials 35, 8951–8959. 10.1016/j.biomaterials.2014.07.012 25085859 PMC4364030

[B9] Baruah H. Puthenveetil S. Choi Y. A. Shah S. Ting A. Y. (2008). An engineered aryl azide ligase for site-specific mapping of protein–protein interactions through photo-cross-linking. Angew. Chem. Int. Ed. Engl. 47, 7126–7129. 10.1002/ange.200802088 PMC2745654 18677791

[B10] Basara G. Yue X. Zorlutuna P. (2019). Dual crosslinked gelatin methacryloyl hydrogels for photolithography and 3D printing. Gels 5, 34–48. 10.3390/gels5030034 31277240 PMC6787727

[B11] Basu S. Cunningham L. P. Pins G. D. Bush K. A. Taboada R. Howell A. R. (2005). Multiphoton excited fabrication of collagen matrixes cross-linked by a modified benzophenone dimer: Bioactivity and enzymatic degradation. Biomacromolecules 6, 1465–1474. 10.1021/bm049258y 15877366

[B12] Blasco E. Wegener M. Barner-Kowollik C. (2017). Photochemically driven polymeric network formation: Synthesis and applications, 29. Wiley Online Libr, 15. 10.1002/adma.201604005 28075059

[B13] Bobula T. Bě͗ák J. Buffa R. Moravcová M. Klein P. Židek O. (2015). Solid-state photocrosslinking of hyaluronan microfibres. Carbohydr. Polym. 125, 153–160. 10.1016/j.carbpol.2015.02.027 25857970

[B14] Brinkman W. T. Nagapudi K. Thomas B. S. Chaikof E. L. (2003). Photo-cross-linking of type I collagen gels in the presence of smooth muscle cells: Mechanical properties, cell viability, and function. Biomacromolecules 4, 890–895. 10.1021/bm0257412 12857069

[B15] Bupphathong S. Quiroz C. Huang W. Chung P. F. Tao H. Y. Lin C. H. (2022). Gelatin methacrylate hydrogel for tissue engineering applications-a review on material modifications. Pharm. (Basel). 15, 171–196. 10.3390/ph15020171 PMC8878046 35215284

[B16] Camp C. P. Peterson I. L. Knoff D. S. Melcher L. G. Maxwell C. J. Cohen A. T. (2020). Non-cytotoxic dityrosine photocrosslinked polymeric materials with targeted elastic moduli. Front. Chem. 8, 173. 10.3389/fchem.2020.00173 32232027 PMC7082925

[B17] Cardenas-Daw C. Kroeger A. Schaertl W. Froimowicz P. Landfester K. (2012). Reversible photocycloadditions, a powerful tool for tailoring (nano)materials. Macromol. Chem. Phys. 213, 144–156. 10.1002/macp.201100399

[B18] Chandrasekharan A. Seong K. Y. Yim S. G. Kim S. Seo S. Yoon J. (2019). *In situ* photocrosslinkable hyaluronic acid-based surgical glue with tunable mechanical properties and high adhesive strength. J. Polym. Sci. A Polym. Chem. J. Polym. Sci. Pol. Chem. Chem. 57, 522–530. 10.1002/pola.29290

[B19] Charron P. N. Fenn S. L. Poniz A. Oldinski R. A. (2016). Mechanical properties and failure analysis of visible light crosslinked alginate-based tissue sealants. J. Mech. Behav. Biomed. Mat. 59, 314–321. 10.1016/j.jmbbm.2016.02.003 PMC4860120 26897093

[B20] Chen L. Kenkel S. M. Hsieh P. H. Gryka M. C. Bhargava R. (2020). Freeform three-dimensionally printed microchannels via surface-initiated photopolymerization combined with sacrificial molding. ACS Appl. Mat. 12, 50105–50112. 10.1021/acsami.0c12158 33091299

[B21] Cho I. S. Cho M. O. Li Z. Nurunnabi M. Park S. Y. Kang S. W. (2016). Synthesis and characterization of a new photo-crosslinkable glycol chitosan thermogel for biomedical applications. Carbohydr. Polym. 144, 59–67. 10.1016/j.carbpol.2016.02.029 27083793

[B22] Choi G. Cha H. J. (2019). Recent advances in the development of nature-derived photocrosslinkable biomaterials for 3D printing in tissue engineering. Biomater. Res. 23, 160–166. 10.1186/s40824-019-0168-8 PMC6862824 31827880

[B23] Chou A. I. Nicoll S. B. (2009). Characterization of photocrosslinked alginate hydrogels for nucleus pulposus cell encapsulation. J. Biomed. Mat. Res. 91, 187–194. 10.1002/jbm.a.32191 18785646

[B24] Chou C. Uprety R. Davis L. Chin J. W. Deiters A. (2011). Genetically encoding an aliphatic diazirine for protein photocrosslinking. Chem. Sci. 2, 480–483. 10.1039/c0sc00373e

[B25] Christensen S. K. Chiappelli M. C. Hayward R. C. (2012). Gelation of copolymers with pendent benzophenone photo-cross-linkers. Macromolecules 45, 5237–5246. 10.1021/ma300784d

[B26] Corey E. J. Bass J. D. LeMahieu R. Mitra R. B. (1964). A study of the photochemical reactions of 2-cyclohexenones with substituted olefins. J. Am. Chem. Soc. 86, 5570–5583. 10.1021/ja01078a034

[B27] Correia M. Neves-Petersen M. T. Jeppesen P. B. Gregersen S. Petersen S. B. (2012). UV-Light exposure of insulin: Pharmaceutical implications upon covalent insulin dityrosine dimerization and disulphide bond photolysis. PLoS One 7, e50733. 10.1371/journal.pone.0050733 23227203 PMC3515625

[B28] Cramer N. B. Reddy S. K. O’Brien A. K. Bowman C. N. (2003). Thiol - ene photopolymerization mechanism and rate limiting step changes for various vinyl functional group chemistries. Macromolecules 36, 7964–7969. 10.1021/ma034667s

[B29] Cruise G. M. Hegre O. D. Scharp D. S. Hubbell J. A. (1998). A sensitivity study of the key parameters in the interfacial photopolymerization of poly(ethylene glycol) diacrylate upon porcine islets. Biotechnol. Bioeng. 57, 655–665. 10.1002/(sici)1097-0290(19980320)57:6<655:aid-bit3>3.0.co;2-k 10099245

[B30] Daood U. Iqbal K. Nitisusanta L. I. Fawzy A. S. (2013). Effect of chitosan/riboflavin modification on resin/dentin interface: Spectroscopic and microscopic investigations. J. Biomed. Mat. Res. - Part A. 101 A, 1846–1856. 10.1002/jbm.a.34482 23184366

[B31] Deseke E. Nakatani Y. Ourisson G. (1998). Intrinsic reactivities of amino acids towards photoalkylation with benzophenone - a study preliminary to photolabelling of the transmembrane protein glycophorin A. Eur. J. Org. Chem. 1998, 243–251. 10.1002/(sici)1099-0690(199802)1998:2<243:aid-ejoc243>3.0.co;2-i

[B32] Devaraj N. K. Weissleder R. Hilderbrand S. A. (2008). Tetrazine-based cycloadditions: Application to pretargeted live cell imaging. Bioconjug. Chem. 19, 2297–2299. 10.1021/bc8004446 19053305 PMC2677645

[B33] Dey K. Roy Chowdhury S. Dykstra E. Lu H. P. Shinar R. Shinar J. (2021). Effect of bis-diazirine-mediated photo-crosslinking on polyvinylcarbazole and solution-processed polymer LEDs. ACS Appl. Electron. Mat. 3, 3365–3371. 10.1021/acsaelm.1c00354

[B34] Dong C. M. Wu X. Caves J. Rele S. S. Thomas B. S. Chaikof E. L. (2005). Photomediated crosslinking of C6-cinnamate derivatized type I collagen. Biomaterials 26, 4041–4049. 10.1016/j.biomaterials.2004.10.017 15626450

[B35] Elbert D. L. Hubbell J. A. (2001). Conjugate addition reactions combined with free-radical cross-linking for the design of materials for tissue engineering. Biomacromolecules 2, 430–441. 10.1021/bm0056299 11749203

[B36] Elvin C. M. Brownlee A. G. Huson M. G. Tebb T. A. Kim M. Lyons R. E. (2009). The development of photochemically crosslinked native fibrinogen as a rapidly formed and mechanically strong surgical tissue sealant. Biomaterials 30, 2059–2065. 10.1016/j.biomaterials.2008.12.059 19147224

[B37] Fancy D. A. Kodadek T. (1999). Chemistry for the analysis of protein-protein interactions: Rapid and efficient cross-linking triggered by long wavelength light. Proc. Natl. Acad. Sci. U. S. A. 96, 6020–6024. 10.1073/pnas.96.11.6020 10339534 PMC26828

[B38] Fernández M. Orozco J. (2021). Advances in functionalized photosensitive polymeric nanocarriers. Polym 13, 2464–2506. 10.3390/polym13152464 PMC8348146 34372067

[B39] Fertier L. Koleilat H. Stemmelen M. Giani O. Joly-Duhamel C. Lapinte V. (2013). The use of renewable feedstock in UV-curable materials – a new age for polymers and green chemistry. Prog. Polym. Sci. 38, 932–962. 10.1016/j.progpolymsci.2012.12.002

[B40] Froimowicz P. Klinger D. Landfester K. (2011). Photoreactive nanoparticles as nanometric building blocks for the generation of self-healing hydrogel thin films. Chem. – A Eur. J. 17, 12465–12475. 10.1002/chem.201100685 21938746

[B41] Fu X. Chang Z. (2019). Biogenesis, quality control, and structural dynamics of proteins as explored in living cells via site-directed photocrosslinking. Protein Sci. 28, 1194–1209. 10.1002/pro.3627 31002747 PMC6566533

[B42] Gadek M. Strachota B. Matějka L. (2021). Photosynthesis of polymer networks with controlled properties by dimerization of cinnamoyl groups. Polym. Int. 70, 1225–1233. 10.1002/pi.6185

[B43] Gattás-Asfura K. M. Weisman E. Andreopoulos F. M. Micic M. Muller B. Sirpal S. (2005). Nitrocinnamate-functionalized gelatin: Synthesis and “smart” hydrogel formation via photo-cross-linking. Biomacromolecules 6, 1503–1509. 10.1021/bm049238w 15877371

[B44] Gaudet I. D. Shreiber D. I. (2012). Characterization of methacrylated type-I collagen as a dynamic, photoactive hydrogel. Biointerphases 7, 25. 10.1007/s13758-012-0025-y 22589068 PMC4243547

[B45] Ge S. S. Chen B. Wu Y. Y. Long Q. S. Zhao Y. L. Wang P. Y. (2018). Current advances of carbene-mediated photoaffinity labeling in medicinal chemistry. RSC Adv. 8, 29428–29454. 10.1039/c8ra03538e 35547988 PMC9084484

[B46] Gillespie J. M. (1972). Proteins rich in glycine and tyrosine from keratins. Comp. Biochem. Physiol. B 41, 723–734. 10.1016/0305-0491(72)90085-5 5032168

[B47] Gong D. Lin Q. Shao Z. Chen X. Yang Y. (2020). Preparing 3D-printable silk fibroin hydrogels with robustness by a two-step crosslinking method. RSC Adv. 10, 27225–27234. 10.1039/d0ra04789a 35515806 PMC9055588

[B48] Gramlich W. M. Kim I. L. Burdick J. A. (2013). Synthesis and orthogonal photopatterning of hyaluronic acid hydrogels with thiol-norbornene chemistry. Biomaterials 34, 9803–9811. 10.1016/j.biomaterials.2013.08.089 24060422 PMC3830935

[B49] Greene T. Lin C. C. (2015). Modular cross-linking of gelatin-based thiol-norbornene hydrogels for *in vitro* 3D culture of hepatocellular carcinoma cells. ACS Biomater. Sci. Eng. 1, 1314–1323. 10.1021/acsbiomaterials.5b00436 33429678

[B50] Guo K. Wang H. Li S. Zhang H. Li S. Zhu H. (2021). Collagen-based thiol-norbornene photoclick bio-ink with excellent bioactivity and printability. ACS Appl. Mat. Interfaces. 13, 7037–7050. 10.1021/acsami.0c16714 33517661

[B51] Gupta P. Trenor S. R. Long T. E. Wilkes G. L. (2004). *In situ* photo-cross-linking of cinnamate functionalized poly(methyl methacrylate-co-2-hydroxyethyl acrylate) fibers during electrospinning. Macromolecules 37, 9211–9218. 10.1021/ma048844g

[B52] Han C. Zhang H. Wu Y. He X. Chen X. (2020). Dual-crosslinked hyaluronan hydrogels with rapid gelation and high injectability for stem cell protection. Sci. Rep. 10, 14997–7. 10.1038/s41598-020-71462-4 32929113 PMC7490415

[B53] Hao Y. Shih H. Muňoz Z. Kemp A. Lin C. C. (2014). Visible light cured thiol-vinyl hydrogels with tunable degradation for 3D cell culture. Acta Biomater. 10, 104–114. 10.1016/j.actbio.2013.08.044 24021231 PMC3840055

[B54] Haria D. P. König B. (2014). Synthetic applications of eosin Y in photoredox catalysis. Chem. Commun. 50, 6688–6699. 10.1039/c4cc00751d 24699920

[B55] Hasany M. Talebian S. Sadat S. Ranjbar N. Mehrali M. Wallace G. G. (2021). Synthesis, properties, and biomedical applications of alginate methacrylate (ALMA)-based hydrogels: Current advances and challenges. Appl. Mat. Today. 24, 101150–101170. 10.1016/j.apmt.2021.101150

[B56] He Y. Li Y. Sun Y. Zhao S. Feng M. Xu G. (2021). A double-network polysaccharide-based composite hydrogel for skin wound healing. Carbohydr. Polym. 261, 117870–117881. 10.1016/j.carbpol.2021.117870 33766357

[B57] Hill J. R. Robertson A. A. B. (2018). Fishing for drug targets: A focus on diazirine photoaffinity probe synthesis. J. Med. Chem. 61, 6945–6963. 10.1021/acs.jmedchem.7b01561 29683660

[B58] Hoffmann N. (2008). Photochemical reactions as key steps in organic synthesis. Chem. Rev. 108, 1052–1103. 10.1021/cr0680336 18302419

[B59] Hoorick J. V. Dobos A. Markovic M. Gheysens T. Damme L. V. Gruber P. (2020). Thiol-norbornene gelatin hydrogels: Influence of thiolated crosslinker on network properties and high definition 3D printing. Biofabrication 13, 015017. 10.1088/1758-5090/abc95f 33176293

[B60] Houée-Lévin C. Bobrowski K. Horakova L. Karademir B. Schöneich C. Davies M. J. (2015). Exploring oxidative modifications of tyrosine: An update on mechanisms of formation, advances in analysis and biological consequences. Free Radic. Res. 49, 347–373. 10.3109/10715762.2015.1007968 25812585

[B61] Hoyle C. E. Bowman C. N. (2010). Thiol–ene click chemistry, 49. Wiley Online Libr., 1540–1573. 10.1002/anie.200903924 20166107

[B62] Huang R. Choe E. Min D. B. (2004). Kinetics for singlet oxygen formation by riboflavin photosensitization and the reaction between riboflavin and singlet oxygen. J. Food Sci. 69, C726–C732. 10.1111/j.1365-2621.2004.tb09924.x

[B63] Huang Y. C. Liu Z. H. Kuo C. Y. Chen J. P. (2022a). Photo-crosslinked hyaluronic acid/carboxymethyl cellulose composite hydrogel as a dural substitute to prevent post-surgical adhesion. Int. J. Mol. Sci. 23, 6177–6195. 10.3390/ijms23116177 35682853 PMC9181059

[B64] Huang Y. Sun G. Lyu L. Li Y. Li D. Fan Q. (2022b). Dityrosine-inspired photocrosslinking technique for 3D printing of silk fibroin-based composite hydrogel scaffolds. Soft Matter 18, 3705–3712. 10.1039/d1sm01817e 35502755

[B65] Jakubovska J. Tauraitė D. Meškys R. (2018). A versatile method for the UVA-induced cross-linking of acetophenone- or benzophenone-functionalized DNA. Sci. Rep. 8, 16484–16510. 10.1038/s41598-018-34892-9 30405165 PMC6220319

[B66] Jeon O. Bouhadir K. H. Mansour J. M. Alsberg E. (2009). Photocrosslinked alginate hydrogels with tunable biodegradation rates and mechanical properties. Biomaterials 30, 2724–2734. 10.1016/j.biomaterials.2009.01.034 19201462

[B67] Keskin D. Mokabbar T. Pei Y. Rijn V. P. (2018). The relationship between bulk silicone and benzophenone-initiated hydrogel coating properties. Polym. (Basel) 10, 534–548. 10.3390/polym10050534 PMC6415430 30966568

[B68] Kianfar P. Vitale A. Vacche D. S. Bongiovanni R. (2019). Photo-crosslinking of chitosan/poly(ethylene oxide) electrospun nanofibers. Carbohydr. Polym. 217, 144–151. 10.1016/j.carbpol.2019.04.062 31079670

[B69] Kim E. H. Han G. D. Noh S. H. Kim J. W. Lee J. G. Ito Y. (2017). Photo-reactive natural polymer derivatives for medical application. J. Ind. Eng. Chem. 54, 1–13. 10.1016/j.jiec.2017.05.029

[B70] Kim E. Seok J. M. Bae S. B. Park S. A. Park W. H. (2021a). Silk fibroin enhances cytocompatibilty and dimensional stability of alginate hydrogels for light-based three-dimensional bioprinting. Biomacromolecules 22, 1921–1931. 10.1021/acs.biomac.1c00034 33840195

[B71] Kim H. Kang B. Cui X. Lee S. H. Lee K. Cho D. W. (2021b). Light-activated decellularized extracellular matrix-based bioinks for volumetric tissue analogs at the centimeter scale. Adv. Funct. Mat. 31, 2011252. 10.1002/adfm.202011252

[B72] Kim S. H. Hong H. Ajiteru O. Sultan M. T. Lee Y. J. Lee J. S. (2021c). 3D bioprinted silk fibroin hydrogels for tissue engineering. Nat. Protoc. 16, 5484–5532. 10.1038/s41596-021-00622-1 34716451

[B73] Kim T. G. Jeong E. H. Lim S. C. Kim S. H. Kim G. H. Kim S. H. (2009). PMMA-based patternable gate insulators for organic thin-film transistors. Synth. Met. 159, 749–753. 10.1016/j.synthmet.2008.11.027

[B74] Kizilel S. Pérez-Luna V. H. Teymour F. (2004). Photopolymerization of poly (ethylene glycol) diacrylate on eosin-functionalized surfaces. Langmuir 20, 8652–8658. 10.1021/la0496744 15379488

[B75] Ko H. F. Sfeir C. Kumta P. N. (2010). Novel synthesis strategies for natural polymer and composite biomaterials as potential scaffolds for tissue engineering. Philos. Trans. Math. Phys. Eng. Sci. 368, 1981–1997. 10.1098/rsta.2010.0009 PMC2944391 20308112

[B76] Kono H. Uno T. Tsujisaki H. Matsushima T. Tajima K. (2020). Nanofibrillated bacterial cellulose modified with (3-aminopropyl)trimethoxysilane under aqueous conditions: Applications to poly(methyl methacrylate) fiber-reinforced nanocomposites. ACS Omega 5, 29561–29569. 10.1021/acsomega.0c04533 33225187 PMC7676300

[B77] Kuboe S. Yoda M. Ogata A. Kitade Y. Tomari Y. Ueno Y. (2010). Diazirine-containing RNA photocrosslinking probes for the study of siRNA-protein interactions. Chem. Commun. (Camb). 46, 7367. 10.1039/c0cc02450c 20820540

[B78] Kumar H. Ambhorkar P. Foulds I. Golovin K. Kim K. (2022). A kinetic model for predicting imperfections in bioink photopolymerization during visible-light stereolithography printing. Addit. Manuf. 55, 102808. 10.1016/j.addma.2022.102808

[B79] Kurioz Y. Reznikov Y. Tereshchenko O. Gerus I. Buluy O. Ha K. R. (2008). Highly sensitive photoaligning materials on a base of cellulose-cinnamates. Mol. Cryst. Liq. Cryst. 480, 81–90. 10.1080/15421400701825458

[B80] Lago M. A. Rodríguez-Bernaldo de Quirós A. Sendón R. Bustos J. Nieto M. T. Paseiro P. (2015). Photoinitiators: A food safety review. Food Addit. Contam. - Chem. Anal. Control Expo. Risk Assess. 32, 779–798. 10.1080/19440049.2015.1014866 25654751

[B81] Lau T. T. Lee L. Q. P. Leong W. Wang D. A. (2012). Formation of model hepatocellular aggregates in a hydrogel scaffold using degradable genipin crosslinked gelatin microspheres as cell carriers. Biomed. Mat. 7, 065003. 10.1088/1748-6041/7/6/065003 23117748

[B82] Lau T. T. Wang C. Png S. W. Su K. Wang D. A. (2011). Genipin-crosslinked microcarriers mediating hepatocellular aggregates formation and functionalities. J. Biomed. Mat. Res. A 96, 204–211. 10.1002/jbm.a.32975 21105169

[B83] Lee J. Ju M. Cho O. H. Kim Y. Nam K. T. (2019). Tyrosine-rich peptides as a platform for assembly and material synthesis. Adv. Sci. (Weinh). 6, 1801255. 10.1002/advs.201801255 30828522 PMC6382316

[B84] Lee M. W. Tsai H. F. Wen S. M. Huang C. H. (2012). Photocrosslinkable gellan gum film as an anti-adhesion barrier. Carbohydr. Polym. 90, 1132–1138. 10.1016/j.carbpol.2012.06.064 22840050

[B85] Lepage M. L. Simhadri C. Liu C. Takaffoli M. Bi L. Crawford B. (2019). A broadly applicable cross-linker for aliphatic polymers containing C–H bonds. Science 366, 875–878. 10.1126/science.aay6230 31727835

[B86] Li P. Müller M. Chang M. W. Frettlöh M. Schönherr H. (2017). Encapsulation of autoinducer sensing reporter bacteria in reinforced alginate-based microbeads. ACS Appl. Mat. Interfaces. 9, 22321–22331. 10.1021/acsami.7b07166 PMC5741077 28627870

[B87] Li Q. Wang D. Elisseeff J. H. (2003). Heterogeneous-phase reaction of glycidyl methacrylate and chondroitin sulfate: Mechanism of ring-opening-transesterification competition. Macromolecules 36, 2556–2562. 10.1021/ma021190w

[B88] Lim D. W. Nettles D. L. Setton L. A. Chilkoti A. (2008). *In situ* cross-linking of elastin-like polypeptide block copolymers for tissue repair. Biomacromolecules 9, 222–230. 10.1021/bm7007982 18163573 PMC3075888

[B89] Lim J. Choi G. Joo K. I. Cha H. J. Kim J. (2021). Embolization of vascular malformations via *in situ* photocrosslinking of mechanically reinforced alginate microfibers using an optical-fiber-integrated microfluidic device. Adv. Mat. 33, 2006759. 10.1002/adma.202006759 33543521

[B90] Lim K. S. Abinzano F. Bernal P. N. Sanchez A. A. Atienza-Roca P. Otto I. A. (2020a). One-step photoactivation of a dual-functionalized bioink as cell carrier and cartilage-binding glue for chondral regeneration. Adv. Healthc. Mat. 9, 1901792. 10.1002/adhm.201901792 PMC7116266 32324342

[B91] Lim K. S. Galarraga J. H. Cui X. Lindberg G. C. J. Burdick J. A. Woodfield T. B. F. (2020b). Fundamentals and applications of photo-cross-linking in bioprinting. Chem. Rev. 120, 10662–10694. 10.1021/acs.chemrev.9b00812 32302091

[B92] Lin C. C. Ki C. S. Shih H. (2015). Thiol–norbornene photoclick hydrogels for tissue engineering applications. J. Appl. Polym. Sci. 132, 41563. 10.1002/app.41563 25558088 PMC4280501

[B93] Lin C. C. Raza A. Shih H. (2011). PEG hydrogels formed by thiol-ene photo-click chemistry and their effect on the formation and recovery of insulin-secreting cell spheroids. Biomaterials 32, 9685–9695. 10.1016/j.biomaterials.2011.08.083 21924490 PMC3195847

[B94] Liu C. Hua J. Ng P. F. Fei B. (2021). Photochemistry of bioinspired dityrosine crosslinking. J. Mat. Sci. Technol. 63, 182–191. 10.1016/j.jmst.2020.02.086

[B95] Liu J. Qu M. Wang C. Xue Y. Huang H. Chen Q. (2022a). A dual-cross-linked hydrogel patch for promoting diabetic wound healing. Small 18, 2106172. 10.1002/smll.202106172 35319815

[B96] Liu J. Su C. Chen Y. Tian S. Lu C. Huang W. (2022b). Current understanding of the applications of photocrosslinked hydrogels in biomedical engineering. Gels 8, 216–242. 10.3390/gels8040216 35448118 PMC9026461

[B97] Liu Q. Tor Y. (2003). Simple conversion of aromatic amines into azides. Org. Lett. 5, 2571–2572. 10.1021/ol034919+ 12841783

[B98] Liu S. Brunel D. Sun K. Zhang Y. Chen H. Xiao P. (2020). Novel photoinitiators based on benzophenone-triphenylamine hybrid structure for LED photopolymerization. Macromol. Rapid Commun. 41, 2000460. 10.1002/marc.202000460 32959447

[B99] Lu H. D. Soranno D. E. Rodell C. B. Kim I. L. Burdick J. A. (2013). Secondary photocrosslinking of injectable shear-thinning dock-and-lock hydrogels. Adv. Healthc. Mat. 2, 1028–1036. 10.1002/adhm.201200343 23299998

[B100] Maiz-Fernández S. Pérez-Álvarez L. Silván U. Vilas-Vilela J. L. Lanceros-Mendez S. (2022). Photocrosslinkable and self-healable hydrogels of chitosan and hyaluronic acid. Int. J. Biol. Macromol. 216, 291–302. 10.1016/j.ijbiomac.2022.07.004 35798076

[B101] Malcor J. D. Mallein-Gerin F. (2022). Biomaterial functionalization with triple-helical peptides for tissue engineering. Acta Biomater. 148, 1–21. 10.1016/j.actbio.2022.06.003 35675889

[B102] Marizza P. Abrami M. Keller S. S. Posocco P. Laurini E. Goswami K. (2016). Synthesis and characterization of UV photocrosslinkable hydrogels with poly(N-vinyl-2-pyrrolidone): Determination of the network mesh size distribution. Int. J. Polym. Mat. Polym. 65, 516–525. 10.1080/00914037.2015.1129964

[B103] McCall J. D. Anseth K. S. (2012). Thiol-ene photopolymerizations provide a facile method to encapsulate proteins and maintain their bioactivity. Biomacromolecules 13, 2410–2417. 10.1021/bm300671s 22741550 PMC3421966

[B104] Messager L. Portecop N. Hachet E. Lapeyre V. Pignot-Paintrand I. Catargi B. (2013). Photochemical crosslinking of hyaluronic acid confined in nanoemulsions: Towards nanogels with a controlled structure. J. Mat. Chem. B 1, 3369. 10.1039/c3tb20300j 32260927

[B105] Michel S. E. S. Dutertre F. Denbow M. L. Galan M. C. Briscoe W. H. (2019). Facile synthesis of chitosan-based hydrogels and microgels through thiol-ene photoclick cross-linking. ACS Appl. Bio Mat. 2, 3257–3268. 10.1021/acsabm.9b00218 35030768

[B106] Michel S. E. S. Rogers S. E. Briscoe W. H. Galan M. C. (2020). Tunable thiol-ene photo-cross-linked chitosan-based hydrogels for biomedical applications. ACS Appl. Bio Mat. 3, 8075–8083. 10.1021/acsabm.0c01171 35019547

[B107] Mihaila S. M. Gaharwar A. K. Reis R. L. Marques A. P. Gomes M. E. Khademhosseini A. (2012). Photocrosslinkable kappa-carrageenan hydrogels for tissue engineering applications. Adv. Healthc. Mater 2, 895–907. 10.1002/adhm.201200317 23281344

[B108] Min K. I. Kim D. H. Lee H. J. Lin L. Kim D. P. (2018). Direct synthesis of a covalently self-assembled peptide nanogel from a tyrosine-rich peptide monomer and its biomineralized hybrids. Angew. Chem. Int. Ed. Engl. 57, 5630–5634. 10.1002/anie.201713261 29569831

[B109] Min K. I. Yun G. Jang Y. Kim K. R. Ko Y. H. Jang H. S. (2016). Covalent self-assembly and one-step photocrosslinking of tyrosine-rich oligopeptides to form diverse nanostructures. Angew. Chem. Int. Ed. 55, 6925–6928. 10.1002/anie.201601675 27062089

[B110] Mishra P. K. Yoo C. M. Hong E. Rhee H. W. (2020). Photo-crosslinking: An emerging chemical tool for investigating molecular networks in live cells. Chembiochem 21, 924–932. 10.1002/cbic.201900600 31794116

[B111] Mu X. Sahoo J. K. Cebe P. Kaplan D. L. (2020). Photo-crosslinked silk fibroin for 3D printing. Polymers 12, 2936–2953. 10.3390/polym12122936 33316890 PMC7763742

[B112] Mukherjee S. Fang M. Kok W. M. Kapp E. A. Thombare V. J. Huguet R. (2019). Establishing signature fragments for identification and sequencing of dityrosine cross-linked peptides using ultraviolet photodissociation mass spectrometry. Anal. Chem. 91, 12129–12133. 10.1021/acs.analchem.9b02986 31490671

[B113] Mũnoz Z. Shih H. Lin C. C. (2014). Gelatin hydrogels formed by orthogonal thiol–norbornene photochemistry for cell encapsulation. Biomater. Sci. 2, 1063–1072. 10.1039/c4bm00070f 32482001

[B114] Murale D. P. Hong S. C. Haque M. M. Lee J. S. (2017). Photo-affinity labeling (PAL) in chemical proteomics: A handy tool to investigate protein-protein interactions (PPIs). Proteome Sci. 15, 14. 10.1186/s12953-017-0123-3 28652856 PMC5483283

[B115] Musolino S. F. Pei Z. Bi L. DiLabio G. A. Wulff J. E. (2021). Structure-function relationships in aryl diazirines reveal optimal design features to maximize C-H insertion. Chem. Sci. 12, 12138–12148. 10.1039/d1sc03631a 34667579 PMC8457397

[B116] Na K. Shin S. Lee H. Shin D. Baek J. Kwak H. (2018). Effect of solution viscosity on retardation of cell sedimentation in DLP 3D printing of gelatin methacrylate/silk fibroin bioink. J. Ind. Eng. Chem. 61, 340–347. 10.1016/j.jiec.2017.12.032

[B117] Nakayama Y. Matsuda T. (1992). Preparation and characteristics of photocrosslinkable hydrophilic polymer having cinnamate moiety. J. Polym. Sci. Part A Polym. Chem. 30, 2451–2457. 10.1002/pola.1992.080301119

[B118] Nam S. Mooney D. (2021). Polymeric tissue adhesives. Chem. Rev. 121, 11336–11384. 10.1021/acs.chemrev.0c00798 33507740

[B119] Navarro J. Swayambunathan J. Santoro M. Fisher J. (2018). Assessment of the effects of energy density in crosslinking of keratin-based photo-sensitive resin. Int. Semin. Biomed. Eng. 2018, 18144107. 10.1109/sib.2018.8467744

[B120] Nettles D. L. Vail T. P. Morgan M. T. Grinstaff M. W. Setton L. A. (2004). Photocrosslinkable hyaluronan as a scaffold for articular cartilage repair. Ann. Biomed. Eng. 32, 391–397. 10.1023/b:abme.0000017552.65260.94 15095813

[B121] Neumann M. G. Schmitt C. C. Goi B. E. Pinto L. F. A. (2013). Photodegradation of polystyrene films containing UV-visible sensitizers. J. Polym. Sci. 2, 39–47.

[B122] Nguyen K. D. Q. Crespo-Ribadeneyra M. Picot O. Colak B. Gautrot J. E. (2021). Ultrafast photo-crosslinking of thiol-norbornene opaque silicone elastomer nanocomposites in air. ACS Appl. Polym. Mat. 3, 5373–5385. 10.1021/acsapm.1c00440

[B123] Noè C. Tonda-Turo C. Chiappone A. Sangermano M. Hakkarainen M. (2020). Light processable starch hydrogels. Polym. (Basel) 12, 1359–1372. 10.3390/polym12061359 PMC7362200 32560332

[B124] Noshadi I. Hong S. Sullivan K. E. Shirzaei Sani E. Portillo-Lara R. Tamayol A. (2017). *In vitro* and *in vivo* analysis of visible light crosslinkable gelatin methacryloyl (GelMA) hydrogels. Biomater. Sci. 5, 2093–2105. 10.1039/c7bm00110j 28805830 PMC5614854

[B125] Occhetta P. Visone R. Russo L. Cipolla L. Moretti M. Rasponi M. (2015). VA-086 methacrylate gelatine photopolymerizable hydrogels: A parametric study for highly biocompatible 3D cell embedding. J. Biomed. Mat. Res. 103, 2109–2117. 10.1002/jbm.a.35346 25294368

[B126] Olson R. A. Korpusik A. B. Sumerlin B. S. (2020). Enlightening advances in polymer bioconjugate chemistry: Light-based techniques for grafting to and from biomacromolecules. Chem. Sci. 11, 5142–5156. 10.1039/d0sc01544j 34122971 PMC8159357

[B127] Ono K. Ishihara M. Ozeki Y. Deguchi H. Sato M. Saito Y. (2001). Experimental evaluation of photocrosslinkable chitosan as a biologic adhesive with surgical applications. Surgery 130, 844–850. 10.1067/msy.2001.117197 11685194

[B128] Ono K. Saito Y. Yura H. Ishikawa K. Kurita A. Akaike T. (2000). Photocrosslinkable chitosan as a biological adhesive. J. Biomed. Mat. Res. 49, 289–295. 10.1002/(sici)1097-4636(200002)49:2<289:aid-jbm18>3.0.co;2-m 10571917

[B129] Orelma H. Vuoriluoto M. Johansson L. S. Campbell J. M. Filpponen I. Biesalski M. (2016). Preparation of photoreactive nanocellulosic materials: Via benzophenone grafting. RSC Adv. 6, 85100–85106. 10.1039/c6ra15015b

[B130] Park T. Y. Yang Y. J. Ha D. H. Cho D. W. Cha H. J. (2019). Marine-derived natural polymer-based bioprinting ink for biocompatible, durable, and controllable 3D constructs. Biofabrication 11 (035001), 035001–035013. 10.1088/1758-5090/ab0c6f 30831562

[B131] Partlow B. P. Applegate M. B. Omenetto F. G. Kaplan D. L. (2016). Dityrosine cross-linking in designing biomaterials. ACS Biomater. Sci. Eng*.* 2, 2108–2121. 10.1021/acsbiomaterials.6b00454 33465886

[B132] Pei M. Mao J. Xu W. Zhou Y. Xiao P. (2019). Photocrosslinkable chitosan hydrogels and their biomedical applications. J. Polym. Sci. Part A Polym. Chem. 57, 1862–1871. 10.1002/pola.29305

[B133] Pereira R. F. Barrias C. C. Bártolo P. J. Granja P. L. (2018a). Cell-instructive pectin hydrogels crosslinked via thiol-norbornene photo-click chemistry for skin tissue engineering. Acta Biomater. 66, 282–293. 10.1016/j.actbio.2017.11.016 29128530

[B134] Pereira R. F. Sousa A. Barrias C. C. Bártolo P. J. Granja P. L. (2018b). A single-component hydrogel bioink for bioprinting of bioengineered 3D constructs for dermal tissue engineering. Mat. Horiz. 5, 1100–1111. 10.1039/c8mh00525g

[B135] Perin F. Mota C. Mancini I. Motta A. Maniglio D. (2022). Photo-enzymatic dityrosine crosslinking for bioprinting. Polymer 252, 124941–124945. 10.1016/j.polymer.2022.124941

[B136] Pham N. D. Parker R. B. Kohler J. J. (2013). Photocrosslinking approaches to interactome mapping. Curr. Opin. Chem. Biol. 17, 90–101. 10.1016/j.cbpa.2012.10.034 23149092 PMC3594551

[B137] Popielarz R. Vogt O. (2008). Effect of coinitiator type on initiation efficiency of two-component photoinitiator systems based on eosin. J. Polym. Sci. Part A Polym. Chem. 46, 3519–3532. 10.1002/pola.22688

[B138] Porter G. Suppan P. (1965). Primary photochemical processes in aromatic molecules. Part 12.—excited states of benzophenone derivatives. J. Chem. Soc. Faraday Trans. 61, 1664–1673. 10.1039/tf9656101664

[B139] Raphel J. Parisi-Amon A. Heilshorn S. C. (2012). Photoreactive elastin-like proteins for use as versatile bioactive materials and surface coatings. J. Mat. Chem. 22, 19429–19437. 10.1039/c2jm31768k PMC3449156 23015764

[B140] Raven D. J. Earland C. Little M. (1971). Occurrence of dityrosine in Tussah silk fibroin and keratin. Biochim. Biophys. Acta - Proteins Proteom. 251, 96–99. 10.1016/0005-2795(71)90065-1 5133280

[B141] Reis A. V. Fajardo A. R. Schuquel I. T. A. Guilherme M. R. Vidotti G. J. Rubira A. F. (2009). Reaction of glycidyl methacrylate at the hydroxyl and carboxylic groups of poly(vinyl alcohol) and poly(acrylic acid): Is this reaction mechanism still unclear? J. Org. Chem. 74, 3750–3757. 10.1021/jo900033c 19361172

[B142] Rennert J. Ruggiero E. Rapp J. (1967). The non‐radiative dissipation of excitation energy in solid cinnamic acid by dimer formation, 6. Wiley Online Libr., 29–34.

[B143] Rickett T. A. Amoozgar Z. Tuchek C. A. Park J. Yeo Y. Shi R. (2011). Rapidly photo-cross-linkable chitosan hydrogel for peripheral neurosurgeries. Biomacromolecules 12, 57–65. 10.1021/bm101004r 21128673

[B144] Roberts J. J. Naudiyal P. Lim K. S. Poole-Warren L. A. Martens P. J. (2016). A comparative study of enzyme initiators for crosslinking phenol-functionalized hydrogels for cell encapsulation. Biomater. Res. 20, 30. 10.1186/s40824-016-0077-z 27713832 PMC5050849

[B145] Samani S. Bonakdar S. Farzin A. Hadjati J. Azami M. (2020). A facile way to synthesize a photocrosslinkable methacrylated chitosan hydrogel for biomedical applications. Int. J. Polym. Mat. Polym. Biomater. 70, 730–741. 10.1080/00914037.2020.1760274

[B146] Sando L. Kim M. Colgrave M. L. Ramshaw J. A. M. Werkmeister J. A. Elvin C. M. (2010). Photochemical crosslinking of soluble wool keratins produces a mechanically stable biomaterial that supports cell adhesion and proliferation. J. Biomed. Mat. Res. A 95A, 901–911. 10.1002/jbm.a.32913 20845488

[B147] Sanyal A. (2010). Diels–alder cycloaddition-cycloreversion: A powerful combo in materials design. Macromol. Chem. Phys. 211, 1417–1425. 10.1002/macp.201000108

[B148] Sarkar D. Bera N. Ghosh S. (2020). [2+2] Photochemical cycloaddition in organic synthesis. Eur. J. Org. Chem. 2020, 1310–1326. 10.1002/ejoc.201901143

[B149] Schelkle K. M. Bender M. Beck S. Jeltsch K. F. Stolz S. Zimmermann J. (2016). Photo-cross-linkable polymeric optoelectronics based on the [2 + 2] cycloaddition reaction of cinnamic acid. Macromolecules 49, 1518–1522. 10.1021/acs.macromol.5b02407

[B150] Schulz A. Gepp M. M. Stracke F. von Briesen H. Neubauer J. C. Zimmermann H. (2019). Tyramine-conjugated alginate hydrogels as a platform for bioactive scaffolds. J. Biomed. Mat. Res. A 107, 114–121. 10.1002/jbm.a.36538 PMC6585978 30256518

[B151] Sharma S. Sudhakara P. Singh J. Ilyas R. A. Asyraf M. R. M. Razman M. R. (2021). Critical review of biodegradable and bioactive polymer composites for bone tissue engineering and drug delivery applications. Polym. (Basel) 13, 2623–2687. 10.3390/polym13162623 PMC8399915 34451161

[B152] Shi D. Liu X. Xiang F. Chen M. Yang C. Akashi M. (2009). Studies on preparation and fluorescent properties of a novel photo-sensitive nanoparticle composed of europium ion and cinnamic acid derivative. Macromol. Chem. Phys. 210, 2063–2069. 10.1002/macp.200900209

[B153] Shi D. Matsusaki M. Kaneko T. Akashi M. (2008). Photo-cross-linking and cleavage induced reversible size change of bio-based nanoparticles. Macromolecules 41, 8167–8172. 10.1021/ma800648e

[B154] Shih H. Lin C. C. (2013). Visible-light-mediated thiol-ene hydrogelation using eosin-y as the only photoinitiator. Macromol. Rapid Commun. 34, 269–273. 10.1002/marc.201200605 23386583

[B155] Singh J. Steele T. W. J. Lim S. (2021). Fibrillated bacterial cellulose liquid carbene bioadhesives for mimicking and bonding oral cavity surfaces. J. Mat. Chem. B 10, 2570–2583. 10.1039/d1tb02044g 34981107

[B156] Sinha Roy P. Mention M. M. Turner M. A. P. Brunissen F. Stavros V. G. Garnier G. (2021). Bio-based photo-reversible self-healing polymer designed from lignin. Green Chem. 23, 10050–10061. 10.1039/d1gc02957f

[B157] Smeds K. A. Pfister-Serres A. Miki D. Dastgheib K. Inoue M. Hatchell D. L. (2001). Photocrosslinkable polysaccharides for *in situ* hydrogel formation. J. Biomed. Mat. Res. 54, 115–121. 10.1002/1097-4636(200101)54:1<115:aid-jbm14>3.0.co;2-q 11077410

[B158] Sogawa H. Katashima T. Numata K. (2020). A covalently crosslinked silk fibroin hydrogel using enzymatic oxidation and chemoenzymatically synthesized copolypeptide crosslinkers consisting of a GPG tripeptide motif and tyrosine: Control of gelation and resilience. Polym. Chem. 11, 3152–3161. 10.1039/d0py00187b

[B159] Soucy J. R. Sani E. S. Lara R. P. Diaz D. Dias F. Weiss A. S. (2018). Photocrosslinkable gelatin/tropoelastin hydrogel adhesives for peripheral nerve repair. Tissue. Eng. Part. A. 24, 17–18. 10.1089/ten.tea.2017.0502 PMC6150941 29580168

[B160] Sun G. Zhang X. Shen Y. I. Sebastian R. Dickinson L. E. Fox-Talbot K. (2011). Dextran hydrogel scaffolds enhance angiogenic responses and promote complete skin regeneration during burn wound healing. Proc. Natl. Acad. Sci. U. S. A. 108, 20976–20981. 10.1073/pnas.1115973108 22171002 PMC3248550

[B161] Sundaramurthi D. Krishnan U. M. Sethuraman S. (2014). Electrospun nanofibers as scaffolds for skin tissue engineering. Polym. Rev. 54, 348–376. 10.1080/15583724.2014.881374

[B162] Sydow S. Aniol A. Hadler C. Menzel H. (2019). Chitosan–azide nanoparticle coating as a degradation barrier in multilayered polyelectrolyte drug delivery systems. Biomolecules 9, 573. 10.3390/biom9100573 31590366 PMC6843188

[B163] Tanaka Y. Bond M. R. Kohler J. J. (2008). Photocrosslinkers illuminate interactions in living cells. Mol. Biosyst. 4, 473. 10.1039/b803218a 18493640

[B164] Temel G. Enginol B. Aydin M. Balta D. K. Arsu N. (2011). Photopolymerization and photophysical properties of amine linked benzophenone photoinitiator for free radical polymerization. J. Photochem. Photobiol. A Chem. 219, 26–31. 10.1016/j.jphotochem.2011.01.012

[B165] Truong M. Y. Dutta N. K. Choudhury N. R. Kim M. Elvin C. M. Nairn K. M. (2011). The effect of hydration on molecular chain mobility and the viscoelastic behavior of resilin-mimetic protein-based hydrogels. Biomaterials 32, 8462–8473. 10.1016/j.biomaterials.2011.07.064 21868089

[B166] Valdes-Aguilera O. Pathak C. P. Shi J. Watson D. Neckers D. C. (1992). Photopolymerization studies using visible light photoinitiators. Macromolecules 25, 541–547. 10.1021/ma00028a008

[B167] Wang J. Yang J. Atif M. Bongiovanni R. Li G. Xue Z. (2018). One-component photoinitiator based on benzophenone and sesamol. Polym. Adv. Technol. 29, 2264–2272. 10.1002/pat.4337

[B168] Wang L. Li J. Zhang D. Ma S. Zhang J. Gao F. (2020). Dual-enzymatically crosslinked and injectable hyaluronic acid hydrogels for potential application in tissue engineering. Rsc. Adv. 10, 2870–2876. 10.1039/c9ra09531d 35496102 PMC9048911

[B169] West A. V. Muncipinto G. Wu H. Y. Huang A. C. Labenski M. T. Jones L. H. (2021). Labeling preferences of diazirines with protein biomolecules. J. Am. Chem. Soc. 143, 6691–6700. 10.1021/jacs.1c02509 33876925 PMC11647638

[B170] Wong J. Kaastrup K. Aguirre-Soto A. Sikes H. D. (2015). A quantitative analysis of peroxy-mediated cyclic regeneration of eosin under oxygen-rich photopolymerization conditions. Polymer 69, 169–177. 10.1016/j.polymer.2015.05.043

[B171] Wu H. Kohler J. (2019). Photocrosslinking probes for capture of carbohydrate interactions. Curr. Opin. Chem. Biol. 53, 173–182. 10.1016/j.cbpa.2019.09.002 31706134 PMC7192017

[B172] Wu Y. Hisada K. Maeda S. Sasaki T. Sakurai K. (2007). Fabrication and structural characterization of the Langmuir–Blodgett films from a new chitosan derivative containing cinnamate chromophores. Carbohydr. Polym. 68, 766–772. 10.1016/j.carbpol.2006.08.016

[B173] Xue T. Li Y. Zhao X. Nie J. Zhu X. (2021). A facile synthesized benzophenone schiff-base ligand as efficient type II visible light photoinitiator. Prog. Org. Coat. 157, 106329. 10.1016/j.porgcoat.2021.106329

[B174] Yagci Y. Jockusch S. Turro N. J. (2010). Photoinitiated polymerization: Advances, challenges, and opportunities. Macromolecules 43, 6245–6260. 10.1021/ma1007545

[B175] Yano S. Iwase T. Shibata M. Miyamoto Y. Shimasaki T. Teramoto N. (2020). Synthesis of photocrosslinkable copolymers of cinnamoyl group-modified methacrylate and 2-hydroxyethyl methacrylate, and fibroblast cell growth on their thin films. J. Photopolym. Sci. Technol. 32, 823–833. 10.2494/photopolymer.32.823

[B176] Ye K. Sinawang P. D. Tok A. I. Y. Marks R. S. (2018). Photoinducible silane diazirine as an effective crosslinker in the construction of a chemiluminescent immunosensor targeting a model E. coli analyte. Sens. Actuators. B Chem. 256, 234–242. 10.1016/j.snb.2017.10.058

[B177] Yu W. Baskin J. M. (2022). Photoaffinity labeling approaches to elucidate lipid–protein interactions. Curr. Opin. Chem. Biol. 69, 102173. 10.1016/j.cbpa.2022.102173 35724595

[B178] Yue K. Li X. Schrobback K. Sheikhi A. Annabi N. Leijten J. (2017). Structural analysis of photocrosslinkable methacryloyl-modified protein derivatives. Biomaterials 139, 163–171. 10.1016/j.biomaterials.2017.04.050 28618346 PMC5845859

[B179] Yuk H. Zhang T. Parada G. A. Liu X. Zhao X. (2016). Skin-inspired hydrogel-elastomer hybrids with robust interfaces and functional microstructures. Nat. Commun. 7, 12028. 10.1038/ncomms12028 27345380 PMC4931236

[B180] Zhang R. Chu F. Hu Y. Hu H. Hu Y. Liu H. (2020). Preparation of photo-crosslinking starch colloidal particles. Starch/Staerke. 72, 1900175–1900176. 10.1002/star.201900175

[B181] Zhao W. Jin X. Cong Y. Liu Y. Fu J. (2013). Degradable natural polymer hydrogels for articular cartilage tissue engineering. J. Chem. Technol. Biotechnol. 88, 327–339. 10.1002/jctb.3970

[B182] Zhou L. Shi H. Li Z. He C. (2020). Recent advances in complex coacervation design from macromolecular assemblies and emerging applications. Macromol. Rapid. Commun. 41, 2000149. 10.1002/marc.202000149 32431012

[B183] Zhu W. Ma D. (2004). Synthesis of aryl azides and vinyl azides via proline-promoted CuI-catalyzed coupling reactions. Chem. Commun. 4, 888–889. 10.1039/b400878b 15045114

